# Experience of Using Electronic Inhaler Monitoring Devices for Patients With Chronic Obstructive Pulmonary Disease or Asthma: Systematic Review of Qualitative Studies

**DOI:** 10.2196/57645

**Published:** 2025-05-16

**Authors:** Jilong Duan, Xia Chen, Di Fan, Haikun Jiang, Xue Zhang, Wenyue Zhang, Zhiping Liu, Hongyan Lu

**Affiliations:** 1 Department of Nursing, Ningxia Medical University, Yinchuan, China; 2 Department of Nursing, General Hospital of Ningxia Medical University, No. 804 Shengli Street, Xingqing District, Yinchuan, 750003, China, 86 0951-6744622

**Keywords:** asthma, drug inhalation, electronic monitoring devices, chronic obstructive pulmonary disease, qualitative research, systematic review

## Abstract

**Background:**

Electronic inhaler monitoring devices (EIMDs) can enhance medication adherence in patients with chronic obstructive pulmonary disease (COPD) and asthma, yet patient perceptions and experiences with these devices vary widely. A systematic qualitative synthesis is required to comprehensively understand patient perspectives on EIMDs, to lay the foundation for developing strategies to improve patient compliance.

**Objective:**

This study aims to systematically evaluate qualitative studies on the experiences of patients with COPD and asthma using EIMDs, providing insights to support their clinical application and improve patient engagement.

**Methods:**

This review synthesized qualitative data from reports found through a systematic search of PubMed, Web of Science, CINAHL, Embase, Cochrane Library, and PsycInfo from January 1983 to July 2024. The reports assessed patient experiences with EIMDs for COPD and asthma. The quality of the included reports was appraised using the Critical Appraisal Skills Program criteria developed by the Centre for Evidence-Based Medicine, University of Oxford, UK.

**Results:**

A total of 7 reports were included, encompassing data from 44 patients with COPD and 146 with asthma. Findings were organized into 9 sub-themes and 3 themes: positive experiences with EIMDs (usability and easy acceptance, enhanced self-management); stresses and challenges of using these devices (negative emotional stress, device trust issues, social difficulties, economic burdens, and technical challenges); and patient expectations from these devices (expectations related to device construction and function and external support).

**Conclusions:**

Patients have positive experiences using electronic monitoring devices for inhalation devices but also face various social, psychological, and technical challenges. Health care workers should consider patient experiences with EIMDs to tailor these devices to patient needs, ultimately enhancing device acceptance and adherence. Further research should focus on increasing EIMDs convenience and usability for patients with COPD and asthma.

## Introduction

Respiratory diseases pose a global health challenge, with chronic obstructive pulmonary disease (COPD) and asthma among the most prevalent types [1]. These conditions result in significant morbidity and mortality worldwide, imposing a substantial and growing health burden [2,3].

While severe respiratory diseases are often incurable, inhaled medications can prevent acute exacerbations [4]. Inhalation therapy, acting directly on the lungs, offers advantages such as rapid onset, low dosage requirements, and minimal side effects [5], making it the primary treatment approach for respiratory conditions like COPD and asthma [6,7].

The efficacy of inhalation therapy relies on the correct use of inhalers [8,9], yet many patients with COPD and asthma struggle with proper technique. Studies report that 4%‐94% of patients do not use inhalation devices correctly [10], which limits the drug’s effectiveness and can lead to poor disease control [11].

Historically, health care providers often use checklists to assess the effectiveness of a patient’s inhalation technique [12]. Although checklists are cost-effective and easy to administer [13], they depend on the skill and knowledge of health care professionals [14,15]. Plaza et al’s [16] questionnaire survey of 1514 practicing physicians and Giner et al’s [17] cross-sectional study of 1496 nurses both found that only approximately 14% of them possessed adequate knowledge about inhalation therapy, suggesting widespread problems with the use of inhalation equipment and a lack of health literacy related to inhalation techniques among health care professionals. This gap in knowledge hinders the ability of medical staff to consistently evaluate the effectiveness of inhalation therapy through standardized assessment tools, thereby compromising the reliability of these evaluations.

A solution to the above problem is electronic monitoring [18]. The International Healthcare Membership Organization defines eHealth as the practice of health care where health care professionals, with the assistance of information engineers, using electronic information or communication technologies to provide health care services and information to patients. eHealth includes forms of applications such as telemedicine, mobile health (mHealth), electronic testing devices, and social media software [19]. Electronic inhaler monitoring devices (EIMDs), also known as smart inhalers, can objectively monitor the adherence and inhalation technical ability of patients with COPD or asthma through built-in sensors, external mobile apps, and other software or hardware [20], as well as provide objective data feedback to patients and health care professionals [21].

The use of EIMDs can be efficacious in improving medication adherence and inhalation techniques, but patient perceptions and experiences while using these devices differ [22]. A meta-analysis by Garin et al [23], found that compared with traditional care, EIMDs can significantly improve medication compliance and inhalation techniques in patients with COPD and asthma. However, each study included in the meta-analysis differed in its approach to intervention. In addition, several published qualitative studies have reported on the experiences of people with COPD or asthma while using EIMDs [24-26], including patient usability and acceptability of the devices, technical challenges in their application by patients, and their costs. However, significant gaps remain in existing qualitative research findings [25]. Diverse cultural, health care, and educational backgrounds may lead to unique patient experiences with EIMDs across different countries [25,27]. Consequently, isolated qualitative studies cannot provide a comprehensive understanding of patient perspectives on these devices for COPD and asthma. Incorporating patient insights may help enhance the functional development of EIMDs [24].

This systematic review aimed to synthesize qualitative studies to create a more comprehensive understanding of the patient experience with EIMDs for COPD and asthma.

## Methods

### Design

This systematic review used Thomas and Harden’s thematic synthesis approach to identify key themes from qualitative data [25]. This method enables the development of new insights based on previous findings. The review protocol is registered with PROSPERO (International Prospective Register of Systematic Reviews) (CRD42023480463).

### Study Selection Criteria

The inclusion criteria were based on the PICoS (Population, Phenomenon of interest, Context, Types of studies) principle. Population: our study population was adult patients with COPD or asthma aged≥18 years. Phenomenon of interest: this systematic review will explore the experiences and perspectives of patients with COPD or asthma who have received EIMDs for inhalation devices. Context: this systematic review considers patient experiences and perspectives with inhaler EIMDs for COPD or asthma, regardless of whether they live at home, in the hospital, in the community, or in other health care settings, as well as regardless of their cultural backgrounds. Types of studies: this systematic review considers all types of qualitative research as well as the qualitative component of mixed studies. We excluded reports that did not provide patient citations and those that did not provide full texts (eg, conference abstracts), as well as study protocols that had not yet been conducted. In addition, we excluded non-English language reports. Specific inclusion and exclusion criteria are shown in Textbox 1.

Textbox 1. **Review inclusion and exclusion criteria.**

**Inclusion Criteria**

- Reports in which the study population comprised patients with chronic obstructive pulmonary disease (COPD) or asthma.
- Reports in which the patients are≥18 years.
- Reports on patients who have had experience or feelings of using electronic inhaler monitoring devices (EIMDs).
- Qualitative research or the qualitative part of mixed research.
- Reports published in English.

**Exclusion Criteria**

- Unpublished articles that have not been peer-reviewed.
- In order to better evaluate the quality of the authors' interpretation and analysis of the data, reports that did not report citations from patients were excluded.
- If the content of an article involved patients with COPD or asthma but did not analyze the data of these patients, the article will be excluded.
- Conference abstracts, quantitative studies, literature reviews, and reports that have not yet been conducted will also be excluded.

### Search Strategy

A comprehensive search was conducted in 6 databases—PubMed, Web of Science, CINAHL, Embase, Cochrane Library, and PsycInfo—to identify qualitative studies on the experiences of patients with COPD or asthma using EIMDs. Electronic monitoring equipment for inhalation devices was first reported in 1983 [28]. To maximize the inclusion of relevant reports, this search covered publications from January 1983 through July 2024. The search terms were formulated according to the PICoS principles and included key terms such as “Pulmonary Disease, Chronic Obstructive/Asthma”, “Electronic/Monitoring/Sensing/passive monitoring/inhaler monitoring/electronic medication monitor/electronic medication/monitoring sensors/medication monitoring”, “Nebulizers and Vaporizers/Inhalers/Inhalator/Inhalation Device/Administration, Inhalation/Drug Administration, Respiratory/Drug Administration”, and “Inhalation interview*/experience*/qualitative”. The search strategy incorporated trade names of existing EIMDs to ensure comprehensive coverage, as recommended by Garin [23] and Kikidis [29]. Details of the search strategy for each database are provided in Multimedia Appendix 1.

### Study Screening and Data Extraction

A total of 2 researchers (DF, ZPL), trained in evidence-based nursing, independently conducted the screening and data extraction processes, adhering to the established inclusion and exclusion criteria. In cases of disagreement, a third researcher (WYZ) was consulted to reach a consensus. Initially, all retrieved reports were imported into EndNote X9 (Clarivate) for deduplication. Titles and abstracts were reviewed to exclude irrelevant studies, followed by a full-text review to confirm the final set of included reports. Data extracted from each report included author details, publication date, country, research method, study population, primary findings, and patient quotations. When reports included perspectives from broader health care interest groups (eg, physicians, nurses, pharmacists, or respiratory therapists), only patient-specific data were extracted for analysis.

### Assessment of Methodological Quality

The methodological quality of included reports was assessed independently by 2 researchers (XC, HKJ) using the Critical Appraisal Skills Programme (CASP) checklist developed by the Centre for Evidence-Based Medicine at the University of Oxford, UK [30]. This tool, focusing on evaluating the validity, utility, and reliability of qualitative research, comprises 10 items evaluated with “yes,” “no,” or “unclear” responses. The CASP checklist enabled the identification of strengths and limitations in each report.

### Data Analysis

Extracted data were imported into NVivo 11.0 software (QSR International) for thematic synthesis, following the method recommended by Thomas and Harden [31]. This approach is conducive to the development of theoretical and conceptual insights applicable to clinical research planning [32]. For the thematic synthesis, data were merged from the included reports, enabling researchers to identify salient themes from each primary report. The synthesis was conducted in 3 stages: first, the 2 researchers (JLD, XZ) independently coded the extracted data from each report line by line. In the second phase, these initial codes were used to construct “descriptive” themes. Finally, in the third phase, the descriptive themes were iteratively examined, aggregated, and generalized to further form “analytical” themes. Any disagreements between the researchers during this process were resolved through discussion with the third researcher (HYL) to reach a consensus and finalize the findings.

## Results

### Search Results

**Figure 1.**
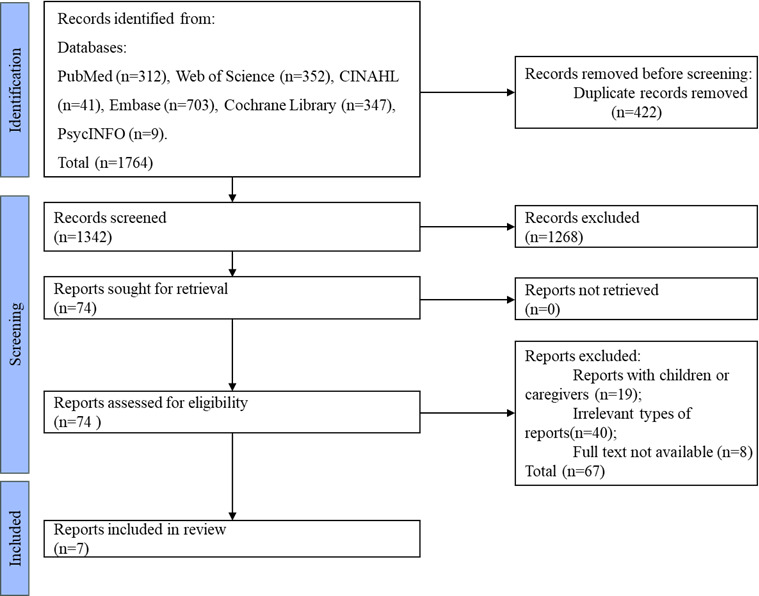
Reports screening flowchart.

The initial database search yielded 1764 articles. After removing 422 duplicates, 1342 articles remained. Following a review of titles and abstracts, 74 potentially relevant articles were selected for further assessment. Full-text analysis led to the final inclusion of 7 articles, all in English. No articles were excluded based on methodological quality assessment. The screening process is illustrated in Figure 1 (Checklist 1).

### Study Characteristics

In total, 7 reports met the inclusion criteria, comprising 4 qualitative studies (57%) and 3 mixed-method studies (43%). Among them 4 of the included reports originated from the United Kingdom (57%), while the remaining 3 were conducted in the Netherlands (14%), Portugal (14%), and Australia (14%). The study population included 44 patients with COPD and 146 patients with asthma. Data were primarily collected through semistructured interviews (n=5), with two reports using focus groups. Additional demographic details are presented in Table 1.

**Table 1.** Basic characteristics of the included reports (n=7).

AuthorYearCountry	Study design	Aim	Participants’ details(Sample sizeGenderMean age or age range)	Data collection methodsData analysis methods	Main results
Van et al [24]2023Netherlands	Qualitative study	Identification of expected facilitators and barriers related to the implementation of smart inhalers	Patients with asthma (n=9) 9 female Mean age 34.7 (13.3)	Focus group Inductive approach	5 themes: Perceived benefits; usability Feasibility Payment and reimbursement Data security and ownership
Hesso et al [33]2023England	Mixed Study	Understanding patients’ perceptions and acceptability of EIMDs	Patients with COPD (n=12); Patients with asthma (n=6) 10 female; 8 male Mean (SD) age: 64.5 (20.3) years	Semistructured interviews Inductive or deductive approaches	4 themes: Acceptability of EIMDs technology Patients misconceptions about the use of EIMDs Acceptability of personalized EIMDs feedback Positive perceptions of tailored consultations
Hui et al [34]2022England	Mixed Study	Understanding patient preferences for documenting asthma and the difficulties encountered in connecting EIMDs to the system to record data	Patients with asthma (n=8) 4 female; 4 male Age range: 26‐65 years	Semistructured interviews Framework analysis	4 themes: performance expectations; effort expectations; social impacts; facilitating conditions
Adejumo et al [25]2022England	Qualitative study	Understanding patients’ perceptions and experiences with EIMDs	Patients with asthma (n=28) 19 female; 9 male Mean age IQR: 46.7 (33.5, 54.2) years	Semistructured interviews Framework approach	5 themes: Participants’ experiences of asthma Participants’ experiences of asthma treatment Participants’ experiences of involvement in research and use of EIMDs Future applications of EIMDs - potential improvements and uses Future applications of EIMDs - desirability, ethics and wider implications
Jácome et al [35]2021Portugal	Mixed Study	Understanding the shortcomings of EIMDs and their adapted applications and suggestions for improvement	Patients with asthma (n=77) Unclear Unclear	Semistructured interviews Thematic qualitative analysis	4 themes: Drug-related characteristics Gamification and social network Symptom monitoring and physician communication Others
Foster et al [26]2017Australia	Qualitative study	To explore patients’ perceptions of barriers, facilitators, usefulness and impact of using the monitor and its reminders	Patients with asthma (n=18) 9 female; 9 male Age range: 18-68 years Mean age: 39 years	Semistructured interviews Thematic approach	3 themes: Feasibility and acceptability Utility and behavioral impact of reminders Sustainability
Kayyali et al [36]2016England	Qualitative study	Understanding the perceptions of people with COPD regarding holistic telemedicine systems and monitoring of inhalation techniques	Patients with COPD (n=32) 15 female; 17 male Unclear	Focus group Inductive or deductive approaches	7 themes: Fragmented care Poor medication adherence Many reasons why patients are not adherent to their medications Poor mental health of patients Limited health care resources available to patients Reported satisfaction Others

### Methodological Quality of the Included Reports

All included reports met the quality standards of the Critical Appraisal Skills Programme (CASP) checklist, with no items marked as “no” or “unclear”. The results of the quality assessment are detailed in Multimedia Appendix 2.

### Synthesis of Research Results

Through a line-by-line coding of the original qualitative data, 41 initial codes were generated. After repeated analysis and comparison, these codes were synthesized into 27 findings, which were organized into nine sub-themes and three overarching themes: (1) positive experiences with EIMDs for patients with COPD or asthma; (2) stresses and challenges associated with using these devices; and (3) patient expectations regarding EIMDs. A summary of the synthesis results is provided in Textbox 2.

Textbox 2. **Thematic analysis and patient quotes from the included reports.**

1. Positive experiences with EIMDs for COPD or asthma patients

- Strong usability and easy acceptance

“It is an easy tool to use” [24]. “It is easy to use” [26].“Recording any inhaler use and reading peak flows would not require much effort for me” [34]; “Both my family doctor and I can see the data that is being recorded” [25]; “The app includes a demonstration on how to use the inhaler with videos” [35].

“I liked it just the way it was” [26]; “It (referring to the chart) is just easier to read” [33]; “It just fits in my pocket” [26]; “I would pay 200-‐400 Australian Dollars for long-time use” [26]*.*

- Improve self-management ability

“With the data, I have a better understanding of my true adherence” [35]; “The feedback data told me I was using the wrong inhalation device” [33]; “It made me realize even more that now I have to use it correctly every morning and evening” [33]. “When data is shared, we can discuss how it is going. Is it possible to change the dose of inhaled medication?” [24]. “I can control it myself now” [26].

2. Stresses and challenges of using EIMDs in patients with COPD or asthma

- Negative emotional stress

“I would like the data generated to be stored on the NHS for security or anonymously stored on the manufacturer’s servers and subject to NHS regulation” [25]; “I think sometimes healthcare professionals receive a lot of useless information or even wrong data” [24]. “I don'’t like being monitored by other people” [24]. “I don'’t need it to remind me because I know I'’m going to take it every day and I'’d rather do it in my own time” [26].

- Lack of trust in EIMDs

“I don'’t think the data it monitors is accurate” [33]; “The default on the system is not the dose I always use” [34]. “It’s best to chat with my doctor to clarify questions and schedule appointments” [35]; “I think contacting the nurse is easiest in general practice” [24].

- Social difficulties

“The buttons on the monitor are too small”, “The monitor is unattractive and monochromatic, I don'’t want to use it in public anymore” [26]; “the reminder goes off and if you go out you have to find a place to hide in a corner to take it out” [26]; “People in the house usually laugh and say ‘you'’re dropping your stuff’, I know they do it to help but I'’ll get there” [26].

- Economic burden

“If it’s very expensive, I don'’t think I'’ll use it because my asthma medication is at a price” [24];

- Device technical challenges

“I need to record some data manually sometimes” [34]. “We face some challenges in setting up the inhaler” [34,36]; “I had two breakdowns while using it and I didn'’t know what to do” [26]. “The app could be smaller” [35]. “Functions like some logins could be simplified” [35].

3. Expectations from EIMDs for patients with COPD or asthma

- Expectations of device construction and function

“Some of the devices are big and not portable” [25]; “If the device could track someone’s movements, what the weather was like, so that you might get a better understanding of asthma symptoms”. “But if it takes more than ten minutes every day then I don'’t have enough time” [24].

- Expectations of external support for COPD or asthma patients

“The monitoring device didn'’t give much advice. Honestly, I think the doctor’s advice was more helpful because she seemed to understand the problem better” [26]. “My fiancé is very, very, very worried about my asthma ...... I take my medication diligently to alleviate his worries” [26]. “I just wish this could be generalized to more patients with asthma” [33]*.*

### Positive Experiences With EIMDs for Patients With COPD or Asthma

Studies indicated that patients with COPD or asthma found EIMDs simple, easy to use, and practical [24,33,34]. Some patients appreciated features like medication reminders [26,33,34], data recording for inhalation techniques [34], data visualization and translation of the collected data, and feedback provision to health care providers; the educational component of accompanying mobile apps was also valued [25,33,35]. High user acceptability was also reported [26], with patients finding data charts clear and easy to interpret [26,33] and display sizes satisfactory [25,26]. Therefore, many patients expressed a willingness to pay for these devices [26].

The devices were found to improve self-management capabilities of patients with COPD or asthma, as they improved medication adherence and allowed monitoring of inhalation technique errors [33], thereby preventing misuse or double dosing and increasing patient awareness of inhalation medication use habits [26,33,35]. In addition, EIMDs facilitated shared decision-making between patients and health care professionals [34], empowering patients to self-manage their health and reduce the burden on health care systems [24,26].

### Stresses and Challenges Associated With EIMDs Use

Patients with COPD or asthma also reported negative emotional stresses with these devices. Around 3 reports reported concerns among patients about data security and loss or incorrect data received by health care professionals [24,25,34]. In addition, some patients felt that certain features of the devices created a feeling of being watched and controlled [24-26].

Trust in using EIMDs was an issue, which resulted in nonadherence to their use, with some patients questioning the accuracy of device-generated measurements and perceived inaccuracies in the displayed information [33,34]. Many patients trusted their health care providers more than their devices [24,35].

Social challenges were also noted. Certain physical characteristics of the devices, such as size, color, and reminder tones, were perceived as barriers to social interaction, as they could attract unwanted attention in public or cause discomfort in relationships with roommates or partners [25,26].

The cost of EIMDs was another obstacle, as the devices were perceived to impose a financial burden on patients. One participant noted, “If it’s very expensive, I don’t think I’ll use it because my asthma medication is at a price” [24].

Technical issues were another challenge. Some patients reported that device functionality was insufficiently developed, leading to inaccuracies and unstable data recording [34]. Operational difficulties arose due to the complex functions of the devices, as mobile apps required substantial storage space and were complicated to navigate [26,34-36]. Incompatibility between monitoring devices and various inhalation devices, as well as between different monitoring device applications, led to patients feeling bored [24].

### Expectations for EIMDs

Patients expressed a desire for more compact, portable, and user-friendly devices that were quick and easy to use. They wanted the devices to offer additional functionality while being time-saving [24,25].

Patients also desired external support guidance, such as family members, doctors, and nurses, when using the devices. Many felt that EIMDs are not a substitute for face-to-face interactions with health care professionals and preferred to rely on specialist nurses and general practitioners for providing support [24,26]. Support from family caregivers was also valued while using EIMDs [26], patients expressed a wish to make the devices easier for other patients to learn to use them [33].

## Discussion

### Principal Findings

This systematic review reveals diverse experiences and perceptions among patients with COPD and asthma regarding electronic inhaler monitoring devices. While many patients report positive experiences, finding these devices helpful in supporting medication adherence and inhalation techniques, they also face notable challenges and concerns. Patients expressed a desire to improve the structural design and functionality of the EIMDs to enhance their ease of use.

Several studies underscore patients’ positive experiences with EIMDs, aligning with the findings in this review [26,37]. Patients generally perceive these devices as versatile and easy to use, offering a range of functional support that enhances their medication adherence and self-management skills. Many view the potential of these devices to aid inhalation techniques positively, expecting that effective use of such features will optimize therapeutic outcomes.

However, the review also indicates that patients often lack a full understanding of these devices’ functionalities, leading to apprehensions about data monitoring, collection, and transmission processes. These concerns are compounded by uncertainties about data security and accuracy, which can erode patient confidence in the devices. As noted by Howard et al [37], features such as timing reminders and device appearance can evoke negative emotional responses, making some patients feel monitored or controlled, thereby reducing social acceptance. Similar themes emerged in this review, as social relationship stressors and technical barriers impacted patients’ perceptions of device usefulness and ease of use. Literature suggests that perceived usefulness and ease of use are pivotal in shaping patient attitudes toward new technologies, which in turn influence user behaviors [38].

Therefore, medical staff should develop standardized protocols for equipment inspection and patient orientation before use. Health care professionals must also remain attentive to patients’ emotional responses, identifying negative reactions promptly and intervening with tailored support to improve patient experiences. By addressing the causes of patient discomfort, medical staff can mitigate barriers to device adoption and enhance patient satisfaction.

Emerging evidence highlights the influence of mHealth technology on patients’ real-life experiences [39]. Despite advancements, current electronic inhaler monitoring devices still lack functionality and compatibility with various inhalers and mobile apps. For example, current devices lack environmental monitoring capabilities, such as air quality feedback, that could provide added value [28,40]. The clinical integration of diverse device-linked mobile apps remains challenging, as does the collection of comprehensive patient data [41]. Design failures are likely when developers overlook patient needs [42]. Thus, clinical staff should regularly collect patient feedback on their expectations for device design and functionality, sharing this input with manufacturers and researchers to drive design and functional upgrades of EIMDs [43].

The future development of electronic inhaler monitoring devices would benefit from a collaborative, interdisciplinary approach. A team inclusive of stakeholders from clinical practice, design, and patient advocacy could apply participatory design methods to develop low-cost, user-friendly devices that meet diverse patient needs, improving both accessibility and acceptance.

Previous research [44] also suggests that family caregivers are supportive of patients adopting innovative mHealth technologies. Patients often rely on family caregivers and peers for assistance with such devices, consistent with our findings. Patients express a desire for family support during device use, underscoring the importance of engaging family members actively in the management of electronic inhaler monitoring devices. Family involvement not only provides oversight but can also promote adherence to prescribed treatment regimens. However, mobile monitoring devices may inadvertently reduce opportunities for direct patient-provider interactions, potentially affecting patient satisfaction and device usage [45]. Some patients believe that electronic monitoring devices cannot fully substitute for in-person communication with health care providers [26]. This review also indicates that patients require ongoing guidance from medical professionals to navigate device use effectively.

To support patient engagement, researchers should develop workflows that integrate electronic inhaler monitoring devices into clinical practice, clearly defining roles and responsibilities for all relevant stakeholders. Establishing a personalized management model that involves patients and their families may ultimately enhance adherence and improve clinical outcomes.

### Limitations

While this thematic review offers insight into variations across individual reports and provides a nuanced understanding of specific issues, certain limitations are unavoidable. The constraints of this systematic review primarily stem from the search strategy and inclusion criteria. Notably, we did not search the websites of companies that manufacture electronic monitoring devices for inhalation, focusing solely on peer-reviewed literature and excluding grey literature. In addition, reports not published in English were excluded, and reports were omitted if they focused exclusively on mobile medical apps without integrating electronic inhaler monitoring devices. Although the reports included represent 4 countries, with over half based in the United Kingdom, introducing potential bias due to limited geographical diversity.

### Conclusion

Through the qualitative synthesis of reports on the experiences and perceptions of COPD and asthma patients using electronic inhaler monitoring devices, this review highlights both positive experiences and significant challenges that impact patient acceptability of these devices. Moving forward, device manufacturers should prioritize equipment and software upgrades that reflect patient expectations and needs. Expanding research on these devices in diverse respiratory patient populations will be essential to ultimately enhance device acceptability and improve patient outcomes.

## Supplementary material

10.2196/57645 Multimedia Appendix 1 Search strategy.

10.2196/57645 Multimedia Appendix 2 Quality assessment form for included studies.

10.2196/57645 Checklist 1 PRISMA checklist

## Abbreviations

CASP: Critical Appraisal Skills Programme
COPD: chronic obstructive pulmonary disease
EIMD: electronic inhaler monitoring device
ENTREQ: Enhancing Transparency in Reporting the Synthesis of Qualitative Research
mHealth: mobile health
NHS: National Health Service
PICoS: Population, Phenomenon of interest, Context, Types of studies
PROSPERO: International Prospective Register of Systematic Reviews
