# Supplementary material for: Experience of Using Electronic Inhaler Monitoring Devices for Patients With Chronic Obstructive Pulmonary Disease or Asthma: Systematic Review of Qualitative Studies
Source: JMIR Mhealth Uhealth. 2025 May 16;13:e57645. doi: 10.2196/57645 (PMC12101605; doi:10.2196/57645)
Supplement: Multimedia Appendix 1 [file mhealth-v13-e57645-s001.doc]

**Multimedia Appendix 1**

**Complete** **search strategy for each database**

**1.1: PubMed database search strategy**

**1.2: Web of Science database search strategy**

**1.3: EMBASE database search strategy**

**1.4: CINAHL database search strategy**

**1.5: PsycINFO database search strategy**

**1.6: The Cochrane Library database search strategy**

**Supplementary file 1.1: PubMed database search strategy**

| Search number | Query | Results |
| --- | --- | --- |
| 48 | (((("Pulmonary Disease, Chronic Obstructive"[Mesh]) OR ((((((((((Chronic Obstructive Lung Disease[Title/Abstract]) OR (Chronic Obstructive Pulmonary Diseases[Title/Abstract])) OR (COAD[Title/Abstract])) OR (COPD[Title/Abstract])) OR (Chronic Obstructive Airway Disease[Title/Abstract])) OR (Chronic Obstructive Pulmonary Disease[Title/Abstract])) OR (Airflow Obstruction, Chronic[Title/Abstract])) OR (Airflow Obstructions, Chronic[Title/Abstract])) OR (Chronic Airflow Obstructions[Title/Abstract])) OR (Chronic Airflow Obstruction[Title/Abstract]))) OR (("Asthma"[Mesh]) OR ((((Asthma[Title/Abstract]) OR (Asthmas[Title/Abstract])) OR (Bronchial Asthma[Title/Abstract])) OR (Asthma, Bronchial[Title/Abstract])))) AND ((((((((((sensor*[Title/Abstract]) OR (passive monitoring[Title/Abstract])) OR (inhaler monitoring[Title/Abstract])) OR (electronic medication monitor[Title/Abstract])) OR (electronic medication[Title/Abstract])) OR (monitoring[Title/Abstract])) OR (monitoring sensors[Title/Abstract])) OR (medication monitoring[Title/Abstract])) AND ((("Nebulizers and Vaporizers"[Mesh]) OR ((((((((((((((Vaporizers[Title/Abstract]) OR (Vaporizer[Title/Abstract])) OR (Inhalers[Title/Abstract])) OR (Inhaler[Title/Abstract])) OR (Inhalators[Title/Abstract])) OR (Inhalator[Title/Abstract])) OR (Nebulizers[Title/Abstract])) OR (Nebulizer[Title/Abstract])) OR (Atomizers[Title/Abstract])) OR (Atomizer[Title/Abstract])) OR (Inhalation Devices[Title/Abstract])) OR (Device, Inhalation[Title/Abstract])) OR (Devices, Inhalation[Title/Abstract])) OR (Inhalation Device[Title/Abstract]))) OR (("Administration, Inhalation"[Mesh]) OR ((((((((((((((Drug Administration, Respiratory[Title/Abstract]) OR (Administration, Respiratory Drug[Title/Abstract])) OR (Respiratory Drug Administration[Title/Abstract])) OR (Inhalation Drug Administration[Title/Abstract])) OR (Drug Administration, Inhalation[Title/Abstract])) OR (Administration, Inhalation Drug[Title/Abstract])) OR (Inhalation Administration[Title/Abstract])) OR (Drug Aerosol Therapy[Title/Abstract])) OR (Aerosol Therapy, Drug[Title/Abstract])) OR (Therapy, Drug Aerosol[Title/Abstract])) OR (Drug Therapy, Aerosol[Title/Abstract])) OR (Aerosol Drug Therapy[Title/Abstract])) OR (Therapy, Aerosol Drug[Title/Abstract])) OR (Inhalation of Drugs[Title/Abstract]))))) OR (((((((((((((((((((((Nebulizer Chronolog[Title/Abstract]) OR (MDI Chronolog[Title/Abstract])) OR (Aerosol Actuation Counter[Title/Abstract])) OR (Turbuhaler Inhalation[Title/Abstract])) OR (Electronic Diskhaler[Title/Abstract])) OR (SmartMist[Title/Abstract])) OR (MDILog[Title/Abstract])) OR (Diskus Adherence Logger[Title/Abstract])) OR (Smart Inhaler Tracker[Title/Abstract])) OR (SmartTrack[Title/Abstract])) OR (SmartDisk[Title/Abstract])) OR (SmartTurbo[Title/Abstract])) OR (SmartFlow[Title/Abstract])) OR (SmartMat[Title/Abstract])) OR (Inhaler Compliance Assessment Device[Title/Abstract])) OR (Asthmapolis[Title/Abstract])) OR (Propeller Health[Title/Abstract])) OR (Chameleon[Title/Abstract])) OR (SmartTouch[Title/Abstract])) OR (MDI Acoustic Actuation Detector[Title/Abstract])) OR (pMDI Datalogger[Title/Abstract])))) AND (((((((((("Qualitative Research"[Mesh]) OR ((Qualitative Research[Title/Abstract]) OR (Research, Qualitative[Title/Abstract]))) OR (("Focus Groups"[Mesh]) OR (((Focus Group[Title/Abstract]) OR (Group, Focus[Title/Abstract])) OR (Groups, Focus[Title/Abstract])))) OR ((("Interviews as Topic"[Mesh]) OR ("Interview" [Publication Type])) OR ((((((((((Interviews, Telephone[Title/Abstract]) OR (Interview, Telephone[Title/Abstract])) OR (Telephone Interview[Title/Abstract])) OR (Telephone Interviews[Title/Abstract])) OR (Group Interviews[Title/Abstract])) OR (Group Interview[Title/Abstract])) OR (Interview, Group[Title/Abstract])) OR (Interviews, Group[Title/Abstract])) OR (Interviewers[Title/Abstract])) OR (Interviewer[Title/Abstract])))) OR ((("Narrative Medicine"[Mesh]) OR ("Personal Narrative" [Publication Type])) OR ((narrative[Title/Abstract]) OR (narration[Title/Abstract])))) OR (("Grounded Theory"[Mesh]) OR ((Grounded Theory[Title/Abstract]) OR (Theory, Grounded[Title/Abstract])))) OR (("Attitude"[Mesh]) OR (((((Attitudes[Title/Abstract]) OR (Sentiment[Title/Abstract])) OR (Sentiments[Title/Abstract])) OR (Opinions[Title/Abstract])) OR (Opinion[Title/Abstract])))) OR (("Hermeneutics"[Mesh]) OR ((hermeneutic[Title/Abstract]) OR (hermeneutics[Title/Abstract])))) OR (("Perception"[Mesh]) OR ((perception[Title/Abstract]) OR (perceptions[Title/Abstract])))) OR (((((((((((((Experience[Title/Abstract]) OR (experiences[Title/Abstract])) OR (perspectives[Title/Abstract])) OR (perspective[Title/Abstract])) OR (beliefs[Title/Abstract])) OR (belief[Title/Abstract])) OR (views[Title/Abstract])) OR (phenomenology[Title/Abstract])) OR (case study[Title/Abstract])) OR (content analysis[Title/Abstract])) OR (descriptive study[Title/Abstract])) OR (thematic analysis[Title/Abstract])) OR (action research[Title/Abstract]))) AND (english[Filter]) | 312 |
| 47 | (((("Pulmonary Disease, Chronic Obstructive"[Mesh]) OR ((((((((((Chronic Obstructive Lung Disease[Title/Abstract]) OR (Chronic Obstructive Pulmonary Diseases[Title/Abstract])) OR (COAD[Title/Abstract])) OR (COPD[Title/Abstract])) OR (Chronic Obstructive Airway Disease[Title/Abstract])) OR (Chronic Obstructive Pulmonary Disease[Title/Abstract])) OR (Airflow Obstruction, Chronic[Title/Abstract])) OR (Airflow Obstructions, Chronic[Title/Abstract])) OR (Chronic Airflow Obstructions[Title/Abstract])) OR (Chronic Airflow Obstruction[Title/Abstract]))) OR (("Asthma"[Mesh]) OR ((((Asthma[Title/Abstract]) OR (Asthmas[Title/Abstract])) OR (Bronchial Asthma[Title/Abstract])) OR (Asthma, Bronchial[Title/Abstract])))) AND ((((((((((sensor*[Title/Abstract]) OR (passive monitoring[Title/Abstract])) OR (inhaler monitoring[Title/Abstract])) OR (electronic medication monitor[Title/Abstract])) OR (electronic medication[Title/Abstract])) OR (monitoring[Title/Abstract])) OR (monitoring sensors[Title/Abstract])) OR (medication monitoring[Title/Abstract])) AND ((("Nebulizers and Vaporizers"[Mesh]) OR ((((((((((((((Vaporizers[Title/Abstract]) OR (Vaporizer[Title/Abstract])) OR (Inhalers[Title/Abstract])) OR (Inhaler[Title/Abstract])) OR (Inhalators[Title/Abstract])) OR (Inhalator[Title/Abstract])) OR (Nebulizers[Title/Abstract])) OR (Nebulizer[Title/Abstract])) OR (Atomizers[Title/Abstract])) OR (Atomizer[Title/Abstract])) OR (Inhalation Devices[Title/Abstract])) OR (Device, Inhalation[Title/Abstract])) OR (Devices, Inhalation[Title/Abstract])) OR (Inhalation Device[Title/Abstract]))) OR (("Administration, Inhalation"[Mesh]) OR ((((((((((((((Drug Administration, Respiratory[Title/Abstract]) OR (Administration, Respiratory Drug[Title/Abstract])) OR (Respiratory Drug Administration[Title/Abstract])) OR (Inhalation Drug Administration[Title/Abstract])) OR (Drug Administration, Inhalation[Title/Abstract])) OR (Administration, Inhalation Drug[Title/Abstract])) OR (Inhalation Administration[Title/Abstract])) OR (Drug Aerosol Therapy[Title/Abstract])) OR (Aerosol Therapy, Drug[Title/Abstract])) OR (Therapy, Drug Aerosol[Title/Abstract])) OR (Drug Therapy, Aerosol[Title/Abstract])) OR (Aerosol Drug Therapy[Title/Abstract])) OR (Therapy, Aerosol Drug[Title/Abstract])) OR (Inhalation of Drugs[Title/Abstract]))))) OR (((((((((((((((((((((Nebulizer Chronolog[Title/Abstract]) OR (MDI Chronolog[Title/Abstract])) OR (Aerosol Actuation Counter[Title/Abstract])) OR (Turbuhaler Inhalation[Title/Abstract])) OR (Electronic Diskhaler[Title/Abstract])) OR (SmartMist[Title/Abstract])) OR (MDILog[Title/Abstract])) OR (Diskus Adherence Logger[Title/Abstract])) OR (Smart Inhaler Tracker[Title/Abstract])) OR (SmartTrack[Title/Abstract])) OR (SmartDisk[Title/Abstract])) OR (SmartTurbo[Title/Abstract])) OR (SmartFlow[Title/Abstract])) OR (SmartMat[Title/Abstract])) OR (Inhaler Compliance Assessment Device[Title/Abstract])) OR (Asthmapolis[Title/Abstract])) OR (Propeller Health[Title/Abstract])) OR (Chameleon[Title/Abstract])) OR (SmartTouch[Title/Abstract])) OR (MDI Acoustic Actuation Detector[Title/Abstract])) OR (pMDI Datalogger[Title/Abstract])))) AND (((((((((("Qualitative Research"[Mesh]) OR ((Qualitative Research[Title/Abstract]) OR (Research, Qualitative[Title/Abstract]))) OR (("Focus Groups"[Mesh]) OR (((Focus Group[Title/Abstract]) OR (Group, Focus[Title/Abstract])) OR (Groups, Focus[Title/Abstract])))) OR ((("Interviews as Topic"[Mesh]) OR ("Interview" [Publication Type])) OR ((((((((((Interviews, Telephone[Title/Abstract]) OR (Interview, Telephone[Title/Abstract])) OR (Telephone Interview[Title/Abstract])) OR (Telephone Interviews[Title/Abstract])) OR (Group Interviews[Title/Abstract])) OR (Group Interview[Title/Abstract])) OR (Interview, Group[Title/Abstract])) OR (Interviews, Group[Title/Abstract])) OR (Interviewers[Title/Abstract])) OR (Interviewer[Title/Abstract])))) OR ((("Narrative Medicine"[Mesh]) OR ("Personal Narrative" [Publication Type])) OR ((narrative[Title/Abstract]) OR (narration[Title/Abstract])))) OR (("Grounded Theory"[Mesh]) OR ((Grounded Theory[Title/Abstract]) OR (Theory, Grounded[Title/Abstract])))) OR (("Attitude"[Mesh]) OR (((((Attitudes[Title/Abstract]) OR (Sentiment[Title/Abstract])) OR (Sentiments[Title/Abstract])) OR (Opinions[Title/Abstract])) OR (Opinion[Title/Abstract])))) OR (("Hermeneutics"[Mesh]) OR ((hermeneutic[Title/Abstract]) OR (hermeneutics[Title/Abstract])))) OR (("Perception"[Mesh]) OR ((perception[Title/Abstract]) OR (perceptions[Title/Abstract])))) OR (((((((((((((Experience[Title/Abstract]) OR (experiences[Title/Abstract])) OR (perspectives[Title/Abstract])) OR (perspective[Title/Abstract])) OR (beliefs[Title/Abstract])) OR (belief[Title/Abstract])) OR (views[Title/Abstract])) OR (phenomenology[Title/Abstract])) OR (case study[Title/Abstract])) OR (content analysis[Title/Abstract])) OR (descriptive study[Title/Abstract])) OR (thematic analysis[Title/Abstract])) OR (action research[Title/Abstract]))) | 328 |
| 46 | ((((((((("Qualitative Research"[Mesh]) OR ((Qualitative Research[Title/Abstract]) OR (Research, Qualitative[Title/Abstract]))) OR (("Focus Groups"[Mesh]) OR (((Focus Group[Title/Abstract]) OR (Group, Focus[Title/Abstract])) OR (Groups, Focus[Title/Abstract])))) OR ((("Interviews as Topic"[Mesh]) OR ("Interview" [Publication Type])) OR ((((((((((Interviews, Telephone[Title/Abstract]) OR (Interview, Telephone[Title/Abstract])) OR (Telephone Interview[Title/Abstract])) OR (Telephone Interviews[Title/Abstract])) OR (Group Interviews[Title/Abstract])) OR (Group Interview[Title/Abstract])) OR (Interview, Group[Title/Abstract])) OR (Interviews, Group[Title/Abstract])) OR (Interviewers[Title/Abstract])) OR (Interviewer[Title/Abstract])))) OR ((("Narrative Medicine"[Mesh]) OR ("Personal Narrative" [Publication Type])) OR ((narrative[Title/Abstract]) OR (narration[Title/Abstract])))) OR (("Grounded Theory"[Mesh]) OR ((Grounded Theory[Title/Abstract]) OR (Theory, Grounded[Title/Abstract])))) OR (("Attitude"[Mesh]) OR (((((Attitudes[Title/Abstract]) OR (Sentiment[Title/Abstract])) OR (Sentiments[Title/Abstract])) OR (Opinions[Title/Abstract])) OR (Opinion[Title/Abstract])))) OR (("Hermeneutics"[Mesh]) OR ((hermeneutic[Title/Abstract]) OR (hermeneutics[Title/Abstract])))) OR (("Perception"[Mesh]) OR ((perception[Title/Abstract]) OR (perceptions[Title/Abstract])))) OR (((((((((((((Experience[Title/Abstract]) OR (experiences[Title/Abstract])) OR (perspectives[Title/Abstract])) OR (perspective[Title/Abstract])) OR (beliefs[Title/Abstract])) OR (belief[Title/Abstract])) OR (views[Title/Abstract])) OR (phenomenology[Title/Abstract])) OR (case study[Title/Abstract])) OR (content analysis[Title/Abstract])) OR (descriptive study[Title/Abstract])) OR (thematic analysis[Title/Abstract])) OR (action research[Title/Abstract])) | 3,066,637 |
| 45 | ((((((((((((Experience[Title/Abstract]) OR (experiences[Title/Abstract])) OR (perspectives[Title/Abstract])) OR (perspective[Title/Abstract])) OR (beliefs[Title/Abstract])) OR (belief[Title/Abstract])) OR (views[Title/Abstract])) OR (phenomenology[Title/Abstract])) OR (case study[Title/Abstract])) OR (content analysis[Title/Abstract])) OR (descriptive study[Title/Abstract])) OR (thematic analysis[Title/Abstract])) OR (action research[Title/Abstract]) | 1,762,870 |
| 44 | ("Perception"[Mesh]) OR ((perception[Title/Abstract]) OR (perceptions[Title/Abstract])) | 732,636 |
| 43 | (perception[Title/Abstract]) OR (perceptions[Title/Abstract]) | 345,851 |
| 42 | "Perception"[Mesh] | 482,717 |
| 41 | ("Hermeneutics"[Mesh]) OR ((hermeneutic[Title/Abstract]) OR (hermeneutics[Title/Abstract])) | 4,220 |
| 40 | (hermeneutic[Title/Abstract]) OR (hermeneutics[Title/Abstract]) | 4,165 |
| 39 | "Hermeneutics"[Mesh] | 579 |
| 38 | ("Attitude"[Mesh]) OR (((((Attitudes[Title/Abstract]) OR (Sentiment[Title/Abstract])) OR (Sentiments[Title/Abstract])) OR (Opinions[Title/Abstract])) OR (Opinion[Title/Abstract])) | 820,020 |
| 37 | ((((Attitudes[Title/Abstract]) OR (Sentiment[Title/Abstract])) OR (Sentiments[Title/Abstract])) OR (Opinions[Title/Abstract])) OR (Opinion[Title/Abstract]) | 277,241 |
| 36 | "Attitude"[Mesh] | 639,780 |
| 35 | ("Grounded Theory"[Mesh]) OR ((Grounded Theory[Title/Abstract]) OR (Theory, Grounded[Title/Abstract])) | 15,756 |
| 34 | (Grounded Theory[Title/Abstract]) OR (Theory, Grounded[Title/Abstract]) | 15,535 |
| 33 | "Grounded Theory"[Mesh] | 2,744 |
| 32 | (("Narrative Medicine"[Mesh]) OR ("Personal Narrative" [Publication Type])) OR ((narrative[Title/Abstract]) OR (narration[Title/Abstract])) | 77,353 |
| 31 | (narrative[Title/Abstract]) OR (narration[Title/Abstract]) | 67,369 |
| 30 | "Narrative Medicine"[Mesh] | 233 |
| 29 | "Personal Narrative" [Publication Type] | 10,143 |
| 28 | (("Interviews as Topic"[Mesh]) OR ("Interview" [Publication Type])) OR ((((((((((Interviews, Telephone[Title/Abstract]) OR (Interview, Telephone[Title/Abstract])) OR (Telephone Interview[Title/Abstract])) OR (Telephone Interviews[Title/Abstract])) OR (Group Interviews[Title/Abstract])) OR (Group Interview[Title/Abstract])) OR (Interview, Group[Title/Abstract])) OR (Interviews, Group[Title/Abstract])) OR (Interviewers[Title/Abstract])) OR (Interviewer[Title/Abstract])) | 134,785 |
| 27 | (((((((((Interviews, Telephone[Title/Abstract]) OR (Interview, Telephone[Title/Abstract])) OR (Telephone Interview[Title/Abstract])) OR (Telephone Interviews[Title/Abstract])) OR (Group Interviews[Title/Abstract])) OR (Group Interview[Title/Abstract])) OR (Interview, Group[Title/Abstract])) OR (Interviews, Group[Title/Abstract])) OR (Interviewers[Title/Abstract])) OR (Interviewer[Title/Abstract]) | 42,798 |
| 26 | "Interviews as Topic"[Mesh] | 66,837 |
| 25 | "Interview" [Publication Type] | 30,785 |
| 24 | ("Focus Groups"[Mesh]) OR (((Focus Group[Title/Abstract]) OR (Group, Focus[Title/Abstract])) OR (Groups, Focus[Title/Abstract])) | 55,922 |
| 23 | ((Focus Group[Title/Abstract]) OR (Group, Focus[Title/Abstract])) OR (Groups, Focus[Title/Abstract]) | 36,264 |
| 22 | "Focus Groups"[Mesh] | 36,288 |
| 21 | ("Qualitative Research"[Mesh]) OR ((Qualitative Research[Title/Abstract]) OR (Research, Qualitative[Title/Abstract])) | 104,640 |
| 20 | (Qualitative Research[Title/Abstract]) OR (Research, Qualitative[Title/Abstract]) | 36,434 |
| 19 | "Qualitative Research"[Mesh] | 84,194 |
| 18 | (((((((((sensor*[Title/Abstract]) OR (passive monitoring[Title/Abstract])) OR (inhaler monitoring[Title/Abstract])) OR (electronic medication monitor[Title/Abstract])) OR (electronic medication[Title/Abstract])) OR (monitoring[Title/Abstract])) OR (monitoring sensors[Title/Abstract])) OR (medication monitoring[Title/Abstract])) AND ((("Nebulizers and Vaporizers"[Mesh]) OR ((((((((((((((Vaporizers[Title/Abstract]) OR (Vaporizer[Title/Abstract])) OR (Inhalers[Title/Abstract])) OR (Inhaler[Title/Abstract])) OR (Inhalators[Title/Abstract])) OR (Inhalator[Title/Abstract])) OR (Nebulizers[Title/Abstract])) OR (Nebulizer[Title/Abstract])) OR (Atomizers[Title/Abstract])) OR (Atomizer[Title/Abstract])) OR (Inhalation Devices[Title/Abstract])) OR (Device, Inhalation[Title/Abstract])) OR (Devices, Inhalation[Title/Abstract])) OR (Inhalation Device[Title/Abstract]))) OR (("Administration, Inhalation"[Mesh]) OR ((((((((((((((Drug Administration, Respiratory[Title/Abstract]) OR (Administration, Respiratory Drug[Title/Abstract])) OR (Respiratory Drug Administration[Title/Abstract])) OR (Inhalation Drug Administration[Title/Abstract])) OR (Drug Administration, Inhalation[Title/Abstract])) OR (Administration, Inhalation Drug[Title/Abstract])) OR (Inhalation Administration[Title/Abstract])) OR (Drug Aerosol Therapy[Title/Abstract])) OR (Aerosol Therapy, Drug[Title/Abstract])) OR (Therapy, Drug Aerosol[Title/Abstract])) OR (Drug Therapy, Aerosol[Title/Abstract])) OR (Aerosol Drug Therapy[Title/Abstract])) OR (Therapy, Aerosol Drug[Title/Abstract])) OR (Inhalation of Drugs[Title/Abstract]))))) OR (((((((((((((((((((((Nebulizer Chronolog[Title/Abstract]) OR (MDI Chronolog[Title/Abstract])) OR (Aerosol Actuation Counter[Title/Abstract])) OR (Turbuhaler Inhalation[Title/Abstract])) OR (Electronic Diskhaler[Title/Abstract])) OR (SmartMist[Title/Abstract])) OR (MDILog[Title/Abstract])) OR (Diskus Adherence Logger[Title/Abstract])) OR (Smart Inhaler Tracker[Title/Abstract])) OR (SmartTrack[Title/Abstract])) OR (SmartDisk[Title/Abstract])) OR (SmartTurbo[Title/Abstract])) OR (SmartFlow[Title/Abstract])) OR (SmartMat[Title/Abstract])) OR (Inhaler Compliance Assessment Device[Title/Abstract])) OR (Asthmapolis[Title/Abstract])) OR (Propeller Health[Title/Abstract])) OR (Chameleon[Title/Abstract])) OR (SmartTouch[Title/Abstract])) OR (MDI Acoustic Actuation Detector[Title/Abstract])) OR (pMDI Datalogger[Title/Abstract])) | 3,471 |
| 17 | ((((((((((((((((((((Nebulizer Chronolog[Title/Abstract]) OR (MDI Chronolog[Title/Abstract])) OR (Aerosol Actuation Counter[Title/Abstract])) OR (Turbuhaler Inhalation[Title/Abstract])) OR (Electronic Diskhaler[Title/Abstract])) OR (SmartMist[Title/Abstract])) OR (MDILog[Title/Abstract])) OR (Diskus Adherence Logger[Title/Abstract])) OR (Smart Inhaler Tracker[Title/Abstract])) OR (SmartTrack[Title/Abstract])) OR (SmartDisk[Title/Abstract])) OR (SmartTurbo[Title/Abstract])) OR (SmartFlow[Title/Abstract])) OR (SmartMat[Title/Abstract])) OR (Inhaler Compliance Assessment Device[Title/Abstract])) OR (Asthmapolis[Title/Abstract])) OR (Propeller Health[Title/Abstract])) OR (Chameleon[Title/Abstract])) OR (SmartTouch[Title/Abstract])) OR (MDI Acoustic Actuation Detector[Title/Abstract])) OR (pMDI Datalogger[Title/Abstract]) | 1,592 |
| 16 | ((((((((sensor*[Title/Abstract]) OR (passive monitoring[Title/Abstract])) OR (inhaler monitoring[Title/Abstract])) OR (electronic medication monitor[Title/Abstract])) OR (electronic medication[Title/Abstract])) OR (monitoring[Title/Abstract])) OR (monitoring sensors[Title/Abstract])) OR (medication monitoring[Title/Abstract])) AND ((("Nebulizers and Vaporizers"[Mesh]) OR ((((((((((((((Vaporizers[Title/Abstract]) OR (Vaporizer[Title/Abstract])) OR (Inhalers[Title/Abstract])) OR (Inhaler[Title/Abstract])) OR (Inhalators[Title/Abstract])) OR (Inhalator[Title/Abstract])) OR (Nebulizers[Title/Abstract])) OR (Nebulizer[Title/Abstract])) OR (Atomizers[Title/Abstract])) OR (Atomizer[Title/Abstract])) OR (Inhalation Devices[Title/Abstract])) OR (Device, Inhalation[Title/Abstract])) OR (Devices, Inhalation[Title/Abstract])) OR (Inhalation Device[Title/Abstract]))) OR (("Administration, Inhalation"[Mesh]) OR ((((((((((((((Drug Administration, Respiratory[Title/Abstract]) OR (Administration, Respiratory Drug[Title/Abstract])) OR (Respiratory Drug Administration[Title/Abstract])) OR (Inhalation Drug Administration[Title/Abstract])) OR (Drug Administration, Inhalation[Title/Abstract])) OR (Administration, Inhalation Drug[Title/Abstract])) OR (Inhalation Administration[Title/Abstract])) OR (Drug Aerosol Therapy[Title/Abstract])) OR (Aerosol Therapy, Drug[Title/Abstract])) OR (Therapy, Drug Aerosol[Title/Abstract])) OR (Drug Therapy, Aerosol[Title/Abstract])) OR (Aerosol Drug Therapy[Title/Abstract])) OR (Therapy, Aerosol Drug[Title/Abstract])) OR (Inhalation of Drugs[Title/Abstract])))) | 1,917 |
| 15 | (("Nebulizers and Vaporizers"[Mesh]) OR ((((((((((((((Vaporizers[Title/Abstract]) OR (Vaporizer[Title/Abstract])) OR (Inhalers[Title/Abstract])) OR (Inhaler[Title/Abstract])) OR (Inhalators[Title/Abstract])) OR (Inhalator[Title/Abstract])) OR (Nebulizers[Title/Abstract])) OR (Nebulizer[Title/Abstract])) OR (Atomizers[Title/Abstract])) OR (Atomizer[Title/Abstract])) OR (Inhalation Devices[Title/Abstract])) OR (Device, Inhalation[Title/Abstract])) OR (Devices, Inhalation[Title/Abstract])) OR (Inhalation Device[Title/Abstract]))) OR (("Administration, Inhalation"[Mesh]) OR ((((((((((((((Drug Administration, Respiratory[Title/Abstract]) OR (Administration, Respiratory Drug[Title/Abstract])) OR (Respiratory Drug Administration[Title/Abstract])) OR (Inhalation Drug Administration[Title/Abstract])) OR (Drug Administration, Inhalation[Title/Abstract])) OR (Administration, Inhalation Drug[Title/Abstract])) OR (Inhalation Administration[Title/Abstract])) OR (Drug Aerosol Therapy[Title/Abstract])) OR (Aerosol Therapy, Drug[Title/Abstract])) OR (Therapy, Drug Aerosol[Title/Abstract])) OR (Drug Therapy, Aerosol[Title/Abstract])) OR (Aerosol Drug Therapy[Title/Abstract])) OR (Therapy, Aerosol Drug[Title/Abstract])) OR (Inhalation of Drugs[Title/Abstract]))) | 49,138 |
| 14 | ("Nebulizers and Vaporizers"[Mesh]) OR ((((((((((((((Vaporizers[Title/Abstract]) OR (Vaporizer[Title/Abstract])) OR (Inhalers[Title/Abstract])) OR (Inhaler[Title/Abstract])) OR (Inhalators[Title/Abstract])) OR (Inhalator[Title/Abstract])) OR (Nebulizers[Title/Abstract])) OR (Nebulizer[Title/Abstract])) OR (Atomizers[Title/Abstract])) OR (Atomizer[Title/Abstract])) OR (Inhalation Devices[Title/Abstract])) OR (Device, Inhalation[Title/Abstract])) OR (Devices, Inhalation[Title/Abstract])) OR (Inhalation Device[Title/Abstract])) | 22,616 |
| 13 | ("Administration, Inhalation"[Mesh]) OR ((((((((((((((Drug Administration, Respiratory[Title/Abstract]) OR (Administration, Respiratory Drug[Title/Abstract])) OR (Respiratory Drug Administration[Title/Abstract])) OR (Inhalation Drug Administration[Title/Abstract])) OR (Drug Administration, Inhalation[Title/Abstract])) OR (Administration, Inhalation Drug[Title/Abstract])) OR (Inhalation Administration[Title/Abstract])) OR (Drug Aerosol Therapy[Title/Abstract])) OR (Aerosol Therapy, Drug[Title/Abstract])) OR (Therapy, Drug Aerosol[Title/Abstract])) OR (Drug Therapy, Aerosol[Title/Abstract])) OR (Aerosol Drug Therapy[Title/Abstract])) OR (Therapy, Aerosol Drug[Title/Abstract])) OR (Inhalation of Drugs[Title/Abstract])) | 34,456 |
| 12 | (((((((((((((Vaporizers[Title/Abstract]) OR (Vaporizer[Title/Abstract])) OR (Inhalers[Title/Abstract])) OR (Inhaler[Title/Abstract])) OR (Inhalators[Title/Abstract])) OR (Inhalator[Title/Abstract])) OR (Nebulizers[Title/Abstract])) OR (Nebulizer[Title/Abstract])) OR (Atomizers[Title/Abstract])) OR (Atomizer[Title/Abstract])) OR (Inhalation Devices[Title/Abstract])) OR (Device, Inhalation[Title/Abstract])) OR (Devices, Inhalation[Title/Abstract])) OR (Inhalation Device[Title/Abstract]) | 17,465 |
| 11 | "Nebulizers and Vaporizers"[Mesh] | 12,883 |
| 10 | (((((((((((((Drug Administration, Respiratory[Title/Abstract]) OR (Administration, Respiratory Drug[Title/Abstract])) OR (Respiratory Drug Administration[Title/Abstract])) OR (Inhalation Drug Administration[Title/Abstract])) OR (Drug Administration, Inhalation[Title/Abstract])) OR (Administration, Inhalation Drug[Title/Abstract])) OR (Inhalation Administration[Title/Abstract])) OR (Drug Aerosol Therapy[Title/Abstract])) OR (Aerosol Therapy, Drug[Title/Abstract])) OR (Therapy, Drug Aerosol[Title/Abstract])) OR (Drug Therapy, Aerosol[Title/Abstract])) OR (Aerosol Drug Therapy[Title/Abstract])) OR (Therapy, Aerosol Drug[Title/Abstract])) OR (Inhalation of Drugs[Title/Abstract]) | 634 |
| 9 | "Administration, Inhalation"[Mesh] | 34,197 |
| 8 | (((((((sensor*[Title/Abstract]) OR (passive monitoring[Title/Abstract])) OR (inhaler monitoring[Title/Abstract])) OR (electronic medication monitor[Title/Abstract])) OR (electronic medication[Title/Abstract])) OR (monitoring[Title/Abstract])) OR (monitoring sensors[Title/Abstract])) OR (medication monitoring[Title/Abstract]) | 1,088,384 |
| 7 | (("Pulmonary Disease, Chronic Obstructive"[Mesh]) OR ((((((((((Chronic Obstructive Lung Disease[Title/Abstract]) OR (Chronic Obstructive Pulmonary Diseases[Title/Abstract])) OR (COAD[Title/Abstract])) OR (COPD[Title/Abstract])) OR (Chronic Obstructive Airway Disease[Title/Abstract])) OR (Chronic Obstructive Pulmonary Disease[Title/Abstract])) OR (Airflow Obstruction, Chronic[Title/Abstract])) OR (Airflow Obstructions, Chronic[Title/Abstract])) OR (Chronic Airflow Obstructions[Title/Abstract])) OR (Chronic Airflow Obstruction[Title/Abstract]))) OR (("Asthma"[Mesh]) OR ((((Asthma[Title/Abstract]) OR (Asthmas[Title/Abstract])) OR (Bronchial Asthma[Title/Abstract])) OR (Asthma, Bronchial[Title/Abstract]))) | 291,453 |
| 6 | ("Asthma"[Mesh]) OR ((((Asthma[Title/Abstract]) OR (Asthmas[Title/Abstract])) OR (Bronchial Asthma[Title/Abstract])) OR (Asthma, Bronchial[Title/Abstract])) | 200,713 |
| 5 | ("Pulmonary Disease, Chronic Obstructive"[Mesh]) OR ((((((((((Chronic Obstructive Lung Disease[Title/Abstract]) OR (Chronic Obstructive Pulmonary Diseases[Title/Abstract])) OR (COAD[Title/Abstract])) OR (COPD[Title/Abstract])) OR (Chronic Obstructive Airway Disease[Title/Abstract])) OR (Chronic Obstructive Pulmonary Disease[Title/Abstract])) OR (Airflow Obstruction, Chronic[Title/Abstract])) OR (Airflow Obstructions, Chronic[Title/Abstract])) OR (Chronic Airflow Obstructions[Title/Abstract])) OR (Chronic Airflow Obstruction[Title/Abstract])) | 107,531 |
| 4 | (((Asthma[Title/Abstract]) OR (Asthmas[Title/Abstract])) OR (Bronchial Asthma[Title/Abstract])) OR (Asthma, Bronchial[Title/Abstract]) | 173,448 |
| 3 | "Asthma"[Mesh] | 143,488 |
| 2 | (((((((((Chronic Obstructive Lung Disease[Title/Abstract]) OR (Chronic Obstructive Pulmonary Diseases[Title/Abstract])) OR (COAD[Title/Abstract])) OR (COPD[Title/Abstract])) OR (Chronic Obstructive Airway Disease[Title/Abstract])) OR (Chronic Obstructive Pulmonary Disease[Title/Abstract])) OR (Airflow Obstruction, Chronic[Title/Abstract])) OR (Airflow Obstructions, Chronic[Title/Abstract])) OR (Chronic Airflow Obstructions[Title/Abstract])) OR (Chronic Airflow Obstruction[Title/Abstract]) | 85,901 |
| 1 | "Pulmonary Disease, Chronic Obstructive"[Mesh] | 67,752 |

**Supplementary file 1.2: Web of Science database search strategy**

| # | Search Query | Results |
| --- | --- | --- |
| 1 | Pulmonary Disease, Chronic Obstructive (Topic) OR Chronic Obstructive Lung Disease (Topic) OR Chronic Obstructive Pulmonary Diseases (Topic) OR COAD (Topic) OR COPD (Topic) OR Chronic Obstructive Airway Disease (Topic) OR Chronic Obstructive Pulmonary Disease (Topic) OR Airflow Obstruction, Chronic (Topic) OR Airflow Obstructions, Chronic (Topic) OR Chronic Airflow Obstructions (Topic) OR Chronic Airflow Obstruction (Topic) | 116722 |
| 2 | Asthma (Topic) OR Asthmas (Topic) OR Bronchial Asthma (Topic) OR Asthma, Bronchial (Topic) | 223289 |
| 3 | #1 OR #2 | 320415 |
| 4 | sensor* (Topic) OR passive monitoring (Topic) OR inhaler monitoring (Topic) OR electronic medication monitor (Topic) OR electronic medication (Topic) OR monitoring (Topic) OR monitoring sensors (Topic) OR medication monitoring (Topic) | 3314115 |
| 5 | Administration, Inhalation (Topic) OR Drug Administration, Respiratory (Topic) OR Administration, Respiratory Drug (Topic) OR Respiratory Drug Administration (Topic) OR Inhalation Drug Administration (Topic) OR Drug Administration, Inhalation (Topic) OR Administration, Inhalation Drug (Topic) OR Inhalation Administration (Topic) OR Drug Aerosol Therapy (Topic) OR Aerosol Therapy, Drug (Topic) OR Therapy, Drug Aerosol (Topic) OR Drug Therapy, Aerosol (Topic) OR Aerosol Drug Therapy (Topic) OR Therapy, Aerosol Drug (Topic) OR Inhalation of Drugs (Topic) | 23403 |
| 6 | Vaporizers and Nebulizers (Topic) OR Vaporizers (Topic) OR Vaporizer (Topic) OR Inhalers (Topic) OR Inhaler (Topic) OR Inhalators (Topic) OR Inhalator (Topic) OR Nebulizers (Topic) OR Nebulizer (Topic) OR Atomizers (Topic) OR Atomizer (Topic) OR Inhalation Devices (Topic) OR Device, Inhalation (Topic) OR Devices, Inhalation (Topic) OR Inhalation Device (Topic) | 32155 |
| 7 | #5 OR #6 | 50971 |
| 8 | #4 AND #7 | 4135 |
| 9 | Nebulizer Chronolog (Topic) OR MDI Chronolog (Topic) OR Aerosol Actuation Counter (Topic) OR Turbuhaler Inhalation (Topic) OR Electronic Diskhaler (Topic) OR SmartMist (Topic) OR MDILog (Topic) OR Diskus Adherence Logger (Topic) OR Smart Inhaler Tracker (Topic) OR SmartTrack (Topic) OR SmartDisk (Topic) OR SmartTurbo (Topic) OR SmartFlow (Topic) OR SmartMat (Topic) OR Inhaler Compliance Assessment Device (Topic) OR Asthmapolis (Topic) OR Propeller Health (Topic) OR Chameleon (Topic) OR SmartTouch (Topic) OR MDI Acoustic Actuation Detector (Topic) OR pMDI Datalogger (Topic) | 5829 |
| 10 | #9 OR #8 | 9887 |
| 11 | Qualitative Research (Topic) OR Research, Qualitative (Topic) | 264553 |
| 12 | Focus Group (Topic) OR Group, Focus (Topic) OR Groups, Focus (Topic) | 434847 |
| 13 | Interviews (Topic) OR Interview (Topic) OR Interviews as Topic (Topic) OR Interviews, Telephone (Topic) OR Interview, Telephone (Topic) OR Telephone Interview (Topic) OR Telephone Interviews (Topic) OR Group Interviews (Topic) OR Group Interview (Topic) OR Interview, Group (Topic) OR Interviews, Group (Topic) OR Interviewers (Topic) OR Interviewer (Topic) | 782726 |
| 14 | Personal Narrative (Topic) OR Narrative Medicine (Topic) OR narrative (Topic) OR narration (Topic) | 258228 |
| 15 | Grounded Theory (Topic) OR Theory, Grounded (Topic) | 117198 |
| 16 | Attitude (Topic) OR Attitudes (Topic) OR Sentiment (Topic) OR Sentiments (Topic) OR Opinions (Topic) OR Opinion (Topic) | 791859 |
| 17 | Hermeneutics (Topic) OR hermeneutic (Topic) OR hermeneutics (Topic) | 20139 |
| 18 | Perception (Topic) OR perception (Topic) OR perceptions (Topic) | 877123 |
| 19 | Experience (Topic) OR experiences (Topic) OR perspectives (Topic) OR perspective (Topic) OR beliefs (Topic) OR belief (Topic) OR views (Topic) OR phenomenology (Topic) OR case study (Topic) OR content analysis (Topic) OR descriptive study (Topic) OR thematic analysis (Topic) OR action research (Topic) | 8943899 |
| 20 | #11 OR #12 OR #13 OR #14 OR #15 OR #16 OR #17 OR #18 OR #19 | 10607979 |
| 21 | #3 AND #10 AND #20 | 368 |
| 22 | #3 AND #10 AND #20 and English (Languages) | 352 |

**Supplementary file 1.3: EMBASE database search strategy**

| No | Query Results | Results |
| --- | --- | --- |
| #45 | #7 AND #19 AND #43 AND [english]/lim | 703 |
| #44 | #7 AND #19 AND #43 | 724 |
| #43 | #22 OR #23 OR #26 OR #30 OR #33 OR #36 OR #39 OR #41 OR #42 | 4,414,401 |
| #42 | 'experience'/exp OR experience OR experiences:ab,ti OR perspectives:ab,ti OR perspective:ab,ti OR beliefs:ab,ti OR belief:ab,ti OR views:ab,ti OR phenomenology:ab,ti OR 'case study':ab,ti OR 'content analysis':ab,ti OR 'descriptive study':ab,ti OR 'thematic analysis':ab,ti OR 'action research':ab,ti | 2,380,116 |
| #41 | 'perception'/exp OR perception OR perceptions:ab,ti | 826,928 |
| #40 | 'perception'/exp | 532,812 |
| #39 | #37 OR #38 | 4,643 |
| #38 | hermeneutic OR hermeneutics:ab,ti | 4,494 |
| #37 | 'hermeneutics'/exp | 813 |
| #36 | #34 OR #35 | 1,224,651 |
| #35 | 'attitude'/exp OR attitude OR attitudes:ab,ti OR sentiment:ab,ti OR sentiments:ab,ti OR opinions:ab,ti OR opinion:ab,ti | 1,224,651 |
| #34 | 'attitude'/exp | 953,767 |
| #33 | #31 OR #32 | 22,774 |
| #32 | 'grounded theory'/exp OR 'grounded theory' OR (grounded AND ('theory'/exp OR theory)) OR 'theory, grounded':ab,ti | 22,774 |
| #31 | 'grounded theory'/exp | 10,438 |
| #30 | #27 OR #28 OR #29 | 79,257 |
| #29 | 'narrative'/exp OR narrative OR narration:ab,ti | 79,257 |
| #28 | 'narrative medicine'/exp | 608 |
| #27 | 'narrative'/exp | 22,483 |
| #26 | #24 OR #25 | 499,679 |
| #25 | 'interview'/exp OR interview OR 'interviews as topic':ab,ti OR 'interviews, telephone':ab,ti OR 'interview, telephone':ab,ti OR 'telephone interview':ab,ti OR 'telephone interviews':ab,ti OR 'group interviews':ab,ti OR 'group interview':ab,ti OR 'interview, group':ab,ti OR 'interviews, group':ab,ti OR interviewers:ab,ti OR interviewer:ab,ti | 499,679 |
| #24 | 'interview'/exp | 375,203 |
| #23 | . 'focus group'/exp OR 'focus group' OR (('focus'/exp OR focus) AND ('group'/exp OR group)) OR 'group, focus':ab,ti OR 'groups, focus':ab,ti | 175,530 |
| #22 | #20 OR #21 | 277,664 |
| #21 | 'qualitative research'/exp OR 'qualitative research' OR (('qualitative'/exp OR qualitative) AND ('research'/exp OR research)) OR 'research, qualitative':ab,ti | 277,664 |
| #20 | 'qualitative research'/exp | 120,604 |
| #19 | #17 OR #18 | 15,394 |
| #18 | 'nebulizer chronolog' OR (('nebulizer'/exp OR nebulizer) AND chronolog) OR 'mdi chronolog':ab,ti OR 'aerosol actuation counter':ab,ti OR 'turbuhaler inhalation':ab,ti OR 'electronic diskhaler':ab,ti OR smartmist:ab,ti OR mdilog:ab,ti OR 'diskus adherence logger':ab,ti OR 'smart inhaler tracker':ab,ti OR smarttrack:ab,ti OR smartdisk:ab,ti OR smartturbo:ab,ti OR smartflow:ab,ti OR smartmat:ab,ti OR 'inhaler compliance assessment device':ab,ti OR asthmapolis:ab,ti OR 'propeller health':ab,ti OR chameleon:ab,ti OR smarttouch:ab,ti OR 'mdi acoustic actuation detector':ab,ti OR 'pmdi datalogger':ab,ti | 1,740 |
| #17 | #8 AND #16 | 13,720 |
| #16 | #11 OR #15 | 304,064 |
| #15 | #12 OR #13 OR #14 | 34,710 |
| #14 | 'vaporizers'/exp OR vaporizers OR vaporizer:ab,ti OR inhalers:ab,ti OR inhaler:ab,ti OR inhalators:ab,ti OR inhalator:ab,ti OR nebulizers:ab,ti OR nebulizer:ab,ti OR atomizers:ab,ti OR atomizer:ab,ti OR 'inhalation devices':ab,ti OR 'device, inhalation':ab,ti OR 'devices, inhalation':ab,ti OR 'inhalation device':ab,ti | 28,522 |
| #13 | 'vaporizer'/exp | 1,689 |
| #12 | 'nebulizer'/exp | 14,007 |
| #11 | #9 OR #10 | 278,212 |
| #10 | 'drug administration, respiratory' OR (('drug'/exp OR drug) AND administration, AND ('respiratory'/exp OR respiratory)) OR 'administration, respiratory drug':ab,ti OR 'respiratory drug administration':ab,ti OR 'inhalation drug administration':ab,ti OR 'drug administration, inhalation':ab,ti OR 'administration, inhalation drug':ab,ti OR 'inhalation administration':ab,ti OR 'drug aerosol therapy':ab,ti OR 'aerosol therapy, drug':ab,ti OR 'therapy, drug aerosol':ab,ti OR 'drug therapy, aerosol':ab,ti OR 'aerosol drug therapy':ab,ti OR 'therapy, aerosol drug':ab,ti OR 'inhalation of drugs':ab,ti | 244,087 |
| #9 | 'inhalational drug administration'/exp | 50,793 |
| #8 | sensor* OR 'passive monitoring':ab,ti OR 'inhaler monitoring':ab,ti OR 'electronic medication monitor':ab,ti OR 'electronic medication':ab,ti OR monitoring:ab,ti OR 'monitoring sensors':ab,ti OR 'medication monitoring':ab,ti | 1,548,224 |
| #7 | #3 OR #6 | 559,255 |
| #6 | #4 OR #5 | 387,936 |
| #5 | 'asthma'/exp OR asthma OR asthmas:ab,ti OR 'bronchial asthma':ab,ti OR 'asthma, bronchial':ab,ti | 387,936 |
| #4 | 'asthma'/exp | 318,667 |
| #3 | #1 OR #2 | 213,022 |
| #2 | 'chronic obstructive lung disease'/exp OR 'chronic obstructive lung disease' OR (chronic AND obstructive AND ('lung'/exp OR lung) AND ('disease'/exp OR disease)) OR 'chronic obstructive pulmonary diseases':ab,ti OR coad:ab,ti OR copd:ab,ti OR 'chronic obstructive airway disease':ab,ti OR 'chronic obstructive pulmonary disease':ab,ti OR 'airflow obstruction, chronic':ab,ti OR 'airflow obstructions, chronic':ab,ti OR 'chronic airflow obstructions':ab,ti OR 'chronic airflow obstruction':ab,ti | 213,022 |
| #1 | 'chronic obstructive lung disease'/exp | 179,463 |

**Supplementary file 1.4: CINAHL database search strategy**

| **#** | Query | Results |
| --- | --- | --- |
| S70 | S9 AND S32 AND S69 | 41 |
| S69 | S36 OR S40 OR S44 OR S49 OR S53 OR S57 OR S61 OR S65 OR S68 | 1,289,378 |
| S68 | S66 OR S67 | 885,194 |
| S67 | AB Experience OR AB experiences OR AB perspectives OR AB perspective OR AB beliefs OR AB belief OR AB views OR AB case study OR AB content analysis OR AB descriptive study OR AB thematic analysis OR AB action research | 719,700 |
| S66 | TI Experience OR TI experiences OR TI perspectives OR TI perspective OR TI beliefs OR TI belief OR TI views OR TI case study OR TI content analysis OR TI descriptive study OR TI thematic analysis OR TI action research | 290,750 |
| S65 | S62 OR S63 OR S64 | 169,483 |
| S64 | AB perception OR AB perceptions | 130,956 |
| S63 | TI perception OR TI perceptions | 54,061 |
| S62 | (MH "Perception") | 31,470 |
| S61 | S58 OR S59 OR S60 | 8,341 |
| S60 | TI phenomenology | 928 |
| S59 | AB phenomenology | 4,456 |
| S58 | (MH "Phenomenology") | 4,277 |
| S57 | S54 OR S55 OR S56 | 150,358 |
| S56 | AB Attitudes OR AB Sentiment OR AB Sentiments OR AB Opinions OR AB Opinion | 119,279 |
| S55 | TI Attitudes OR TI Sentiment OR TI Sentiments OR TI Opinions OR TI Opinion | 46,157 |
| S54 | (MH "Attitude") | 18,261 |
| S53 | S50 OR S51 OR S52 | 22,209 |
| S52 | AB Grounded Theory OR AB Theory, Grounded | 14,095 |
| S51 | TI Grounded Theory OR TI Theory, Grounded | 2,221 |
| S50 | (MH "Grounded Theory") | 18,212 |
| S49 | S45 OR S46 OR S47 OR S48 | 57,946 |
| S48 | AB narrative OR AB narration OR AB Personal Narrative | 41,746 |
| S47 | TI narrative OR TI narration OR TI Personal Narrative | 13,888 |
| S46 | (MH "Narrative Medicine") | 179 |
| S45 | (MH "Narratives") | 20,727 |
| S44 | S41 OR S42 OR S43 | 323,929 |
| S43 | AB Interview OR AB Interviews as Topic OR AB Interviews, Telephone OR AB Interview, Telephone OR AB Telephone Interview OR AB Telephone Interviews OR AB Group Interviews OR AB Group Interview OR AB Interview, Group OR AB Interviews, Group OR AB Interviewers OR AB Interviewer | 224,985 |
| S42 | TI Interview OR TI Interviews as Topic OR TI Interviews, Telephone OR TI Interview, Telephone OR TI Telephone Interview OR TI Telephone Interviews OR TI Group Interviews OR TI Group Interview OR TI Interview, Group OR TI Interviews, Group OR TI Interviewers OR TI Interviewer | 15,325 |
| S41 | (MH "Interviews") | 168,292 |
| S40 | S37 OR S38 OR S39 | 65,421 |
| S39 | AB Focus Group OR AB Group, Focus OR AB Groups, Focus | 46,757 |
| S38 | TI Focus Group OR TI Group, Focus OR TI Groups, Focus | 3,307 |
| S37 | (MH "Focus Groups") | 51,052 |
| S36 | S33 OR S34 OR S35 | 179,301 |
| S35 | AB Qualitative Research OR AB Research, Qualitative OR AB Qualitative Studies | 79,285 |
| S34 | TI Qualitative Research OR TI Research, Qualitative OR TI Qualitative Studies | 32,368 |
| S33 | (MH "Qualitative Studies") | 147,228 |
| S32 | S26 OR S31 | 775 |
| S31 | S27 OR S28 OR S29 OR S30 | 312 |
| S30 | AB SmartFlow OR AB SmartMat OR AB Inhaler Compliance Assessment Device OR AB Asthmapolis OR AB Propeller Health OR AB Chameleon OR AB SmartTouch OR AB MDI Acoustic Actuation Detector OR AB pMDI Datalogger | 179 |
| S29 | TI SmartFlow OR TI SmartMat OR TI Inhaler Compliance Assessment Device OR TI Asthmapolis OR TI Propeller Health OR TI Chameleon OR TI SmartTouch OR TI MDI Acoustic Actuation Detector OR TI pMDI Datalogger | 141 |
| S28 | AB Nebulizer Chronolog OR AB MDI Chronolog OR AB Aerosol Actuation Counter OR AB Turbuhaler Inhalation OR AB Electronic Diskhaler OR AB SmartMist OR AB MDILog OR AB Diskus Adherence Logger OR AB Smart Inhaler Tracker OR AB SmartTrack OR AB SmartDisk OR AB SmartTurbo | 33 |
| S27 | TI Nebulizer Chronolog OR TI MDI Chronolog OR TI Aerosol Actuation Counter OR TI Turbuhaler Inhalation OR TI Electronic Diskhaler OR TI SmartMist OR TI MDILog OR TI Diskus Adherence Logger OR TI Smart Inhaler Tracker OR TI SmartTrack OR TI SmartDisk OR TI SmartTurbo | 7 |
| S26 | S12 AND S25 | 473 |
| S25 | S18 OR S24 | 14,058 |
| S24 | S19 OR S20 OR S21 OR S22 OR S23 | 7,819 |
| S23 | AB Nebulizers OR AB Nebulizer OR AB Atomizers OR AB Atomizer OR AB Device, Inhalation OR AB Devices, Inhalation OR AB Inhalation Device | 1,100 |
| S22 | TI Nebulizers OR TI Nebulizer OR TI Atomizers OR TI Atomizer OR TI Device, Inhalation OR TI Devices, Inhalation OR TI Inhalation Device | 442 |
| S21 | AB Vaporizers OR AB Vaporizer OR AB Inhalers OR AB Inhaler OR AB Inhalators OR AB Inhalator OR AB Inhalation Devices | 2,926 |
| S20 | TI Vaporizers OR TI Vaporizer OR TI Inhalers OR TI Inhaler OR TI Inhalators OR TI Inhalator OR TI Inhalation Devices | 1,853 |
| S19 | (MH "Nebulizers and Vaporizers") | 5,535 |
| S18 | S13 OR S14 OR S15 OR S16 OR S17 | 8,277 |
| S17 | AB Drug Aerosol Therapy OR AB Aerosol Therapy, Drug OR AB Therapy, Drug Aerosol OR AB Drug Therapy, Aerosol OR AB Aerosol Drug Therapy OR AB Therapy, Aerosol Drug OR AB Inhalation of Drugs | 318 |
| S16 | TI Drug Aerosol Therapy OR TI Aerosol Therapy, Drug OR TI Therapy, Drug Aerosol OR TI Drug Therapy, Aerosol OR TI Aerosol Drug Therapy OR TI Therapy, Aerosol Drug OR TI Inhalation of Drugs | 53 |
| S15 | AB Drug Administration, Respiratory OR AB Administration, Respiratory Drug OR AB Respiratory Drug Administration OR AB Inhalation Drug Administration OR AB Drug Administration, Inhalation OR AB Administration, Inhalation Drug OR AB Inhalation Administration | 193 |
| S14 | TI Drug Administration, Respiratory OR TI Administration, Respiratory Drug OR TI Respiratory Drug Administration OR TI Inhalation Drug Administration OR TI Drug Administration, Inhalation OR TI Administration, Inhalation Drug OR TI Inhalation Administration | 38 |
| S13 | (MH "Administration, Inhalation") | 7,903 |
| S12 | S10 OR S11 | 179,484 |
| S11 | AB sensor* OR AB passive monitoring OR AB inhaler monitoring OR AB electronic medication monitor OR AB electronic medication OR AB monitoring OR AB monitoring sensors OR AB medication monitoring | 155,344 |
| S10 | TI sensor* OR TI passive monitoring OR TI inhaler monitoring OR TI electronic medication monitor OR TI electronic medication OR TI monitoring OR TI monitoring sensors OR TI medication monitoring | 48,102 |
| S9 | S4 OR S8 | 78,165 |
| S8 | S5 OR S6 OR S7 | 49,817 |
| S7 | AB Asthma OR AB Asthmas OR AB Bronchial Asthma OR AB Asthma, Bronchial | 29,517 |
| S6 | TI Asthma OR TI Asthmas OR TI Bronchial Asthma OR TI Asthma, Bronchial | 26,688 |
| S5 | (MH "Asthma") | 37,233 |
| S4 | S1 OR S2 OR S3 | 32,567 |
| S3 | AB Chronic Obstructive Lung Disease OR AB Chronic Obstructive Pulmonary Diseases OR AB COAD OR AB COPD OR AB Chronic Obstructive Airway Disease OR AB Chronic Obstructive Pulmonary Disease OR AB Airflow Obstruction, Chronic OR AB Airflow Obstructions, Chronic OR AB Chronic Airflow Obstructions OR AB Chronic Airflow Obstruction | 20,896 |
| S2 | TI Chronic Obstructive Lung Disease OR TI Chronic Obstructive Pulmonary Diseases OR TI COAD OR TI COPD OR TI Chronic Obstructive Airway Disease OR TI Chronic Obstructive Pulmonary Disease OR TI Airflow Obstruction, Chronic OR TI Airflow Obstructions, Chronic OR TI Chronic Airflow Obstructions OR TI Chronic Airflow Obstruction | 18,299 |
| S1 | (MH "Pulmonary Disease, Chronic Obstructive") | 22,043 |

**Supplementary file 1.5: PsycINFO database search strategy**

| # | Query | Results |
| --- | --- | --- |
| S70 | S9 AND S32 AND S69 | 9 |
| S69 | S36 OR S40 OR S44 OR S49 OR S53 OR S57 OR S61 OR S65 OR S68 | 1,869,215 |
| S68 | S66 OR S67 | 1,308,064 |
| S67 | AB Experience OR AB experiences OR AB perspectives OR AB perspective OR AB beliefs OR AB belief OR AB views OR AB case study OR AB content analysis OR AB descriptive study OR AB thematic analysis OR AB action research | 1,222,882 |
| S66 | TI Experience OR TI experiences OR TI perspectives OR TI perspective OR TI beliefs OR TI belief OR TI views OR TI case study OR TI content analysis OR TI descriptive study OR TI thematic analysis OR TI action research | 291,919 |
| S65 | S62 OR S63 OR S64 | 344,153 |
| S64 | AB perception OR AB perceptions | 308,358 |
| S63 | TI perception OR TI perceptions | 108,959 |
| S62 | (MH "Perception") | 13 |
| S61 | S58 OR S59 OR S60 | 15,506 |
| S60 | TI phenomenology | 13,812 |
| S59 | AB phenomenology | 3,346 |
| S58 | (MH "Phenomenology") | 1 |
| S57 | S54 OR S55 OR S56 | 275,879 |
| S56 | AB Attitudes OR AB Sentiment OR AB Sentiments OR AB Opinions OR AB Opinion | 257,328 |
| S55 | TI Attitudes OR TI Sentiment OR TI Sentiments OR TI Opinions OR TI Opinion | 74,929 |
| S54 | (MH "Attitude") | 3 |
| S53 | S50 OR S51 OR S52 | 23,620 |
| S52 | AB Grounded Theory OR AB Theory, Grounded | 23,312 |
| S51 | TI Grounded Theory OR TI Theory, Grounded | 3,100 |
| S50 | (MH "Grounded Theory") | 1 |
| S49 | S45 OR S46 OR S47 OR S48 | 84,457 |
| S48 | AB narrative OR AB narration OR AB Personal Narrative | 80,923 |
| S47 | TI narrative OR TI narration OR TI Personal Narrative | 20,906 |
| S46 | (MH "Narrative Medicine") | 541 |
| S45 | (MH "Narratives") | 8 |
| S44 | S41 OR S42 OR S43 | 342,520 |
| S43 | AB Interview OR AB Interviews as Topic OR AB Interviews, Telephone OR AB Interview, Telephone OR AB Telephone Interview OR AB Telephone Interviews OR AB Group Interviews OR AB Group Interview OR AB Interview, Group OR AB Interviews, Group OR AB Interviewers OR AB Interviewer | 340,213 |
| S42 | TI Interview OR TI Interviews as Topic OR TI Interviews, Telephone OR TI Interview, Telephone OR TI Telephone Interview OR TI Telephone Interviews OR TI Group Interviews OR TI Group Interview OR TI Interview, Group OR TI Interviews, Group OR TI Interviewers OR TI Interviewer | 15,361 |
| S41 | (MH "Interviews") | 15 |
| S40 | S37 OR S38 OR S39 | 52,581 |
| S39 | AB Focus Group OR AB Group, Focus OR AB Groups, Focus | 52,461 |
| S38 | TI Focus Group OR TI Group, Focus OR TI Groups, Focus | 2,228 |
| S37 | (MH "Focus Groups") | 6 |
| S36 | S33 OR S34 OR S35 | 121,195 |
| S35 | AB Qualitative Research OR AB Research, Qualitative OR AB Qualitative Studies | 110,144 |
| S34 | TI Qualitative Research OR TI Research, Qualitative OR TI Qualitative Studies | 23,835 |
| S33 | (MH "Qualitative Studies") | 584 |
| S32 | S26 OR S31 | 344 |
| S31 | S27 OR S28 OR S29 OR S30 | 289 |
| S30 | AB SmartFlow OR AB SmartMat OR AB Inhaler Compliance Assessment Device OR AB Asthmapolis OR AB Propeller Health OR AB Chameleon OR AB SmartTouch OR AB MDI Acoustic Actuation Detector OR AB pMDI Datalogger | 190 |
| S29 | TI SmartFlow OR TI SmartMat OR TI Inhaler Compliance Assessment Device OR TI Asthmapolis OR TI Propeller Health OR TI Chameleon OR TI SmartTouch OR TI MDI Acoustic Actuation Detector OR TI pMDI Datalogger | 159 |
| S28 | AB Nebulizer Chronolog OR AB MDI Chronolog OR AB Aerosol Actuation Counter OR AB Turbuhaler Inhalation OR AB Electronic Diskhaler OR AB SmartMist OR AB MDILog OR AB Diskus Adherence Logger OR AB Smart Inhaler Tracker OR AB SmartTrack OR AB SmartDisk OR AB SmartTurbo | 9 |
| S27 | TI Nebulizer Chronolog OR TI MDI Chronolog OR TI Aerosol Actuation Counter OR TI Turbuhaler Inhalation OR TI Electronic Diskhaler OR TI SmartMist OR TI MDILog OR TI Diskus Adherence Logger OR TI Smart Inhaler Tracker OR TI SmartTrack OR TI SmartDisk OR TI SmartTurbo | 0 |
| S26 | S12 AND S25 | 56 |
| S25 | S18 OR S24 | 735 |
| S24 | S19 OR S20 OR S21 OR S22 OR S23 | 579 |
| S23 | AB Nebulizers OR AB Nebulizer OR AB Atomizers OR AB Atomizer OR AB Device, Inhalation OR AB Devices, Inhalation OR AB Inhalation Device | 83 |
| S22 | TI Nebulizers OR TI Nebulizer OR TI Atomizers OR TI Atomizer OR TI Device, Inhalation OR TI Devices, Inhalation OR TI Inhalation Device | 15 |
| S21 | AB Vaporizers OR AB Vaporizer OR AB Inhalers OR AB Inhaler OR AB Inhalators OR AB Inhalator OR AB Inhalation Devices | 530 |
| S20 | TI Vaporizers OR TI Vaporizer OR TI Inhalers OR TI Inhaler OR TI Inhalators OR TI Inhalator OR TI Inhalation Devices | 115 |
| S19 | (MH "Nebulizers and Vaporizers") | 144 |
| S18 | S13 OR S14 OR S15 OR S16 OR S17 | 170 |
| S17 | AB Drug Aerosol Therapy OR AB Aerosol Therapy, Drug OR AB Therapy, Drug Aerosol OR AB Drug Therapy, Aerosol OR AB Aerosol Drug Therapy OR AB Therapy, Aerosol Drug OR AB Inhalation of Drugs | 96 |
| S16 | TI Drug Aerosol Therapy OR TI Aerosol Therapy, Drug OR TI Therapy, Drug Aerosol OR TI Drug Therapy, Aerosol OR TI Aerosol Drug Therapy OR TI Therapy, Aerosol Drug OR TI Inhalation of Drugs | 6 |
| S15 | AB Drug Administration, Respiratory OR AB Administration, Respiratory Drug OR AB Respiratory Drug Administration OR AB Inhalation Drug Administration OR AB Drug Administration, Inhalation OR AB Administration, Inhalation Drug OR AB Inhalation Administration | 70 |
| S14 | TI Drug Administration, Respiratory OR TI Administration, Respiratory Drug OR TI Respiratory Drug Administration OR TI Inhalation Drug Administration OR TI Drug Administration, Inhalation OR TI Administration, Inhalation Drug OR TI Inhalation Administration | 10 |
| S13 | (MH "Administration, Inhalation") | 0 |
| S12 | S10 OR S11 | 174,231 |
| S11 | AB sensor* OR AB passive monitoring OR AB inhaler monitoring OR AB electronic medication monitor OR AB electronic medication OR AB monitoring OR AB monitoring sensors OR AB medication monitoring | 167,336 |
| S10 | TI sensor* OR TI passive monitoring OR TI inhaler monitoring OR TI electronic medication monitor OR TI electronic medication OR TI monitoring OR TI monitoring sensors OR TI medication monitoring | 33,074 |
| S9 | S4 OR S8 | 10,675 |
| S8 | S5 OR S6 OR S7 | 8,128 |
| S7 | AB Asthma OR AB Asthmas OR AB Bronchial Asthma OR AB Asthma, Bronchial | 7,919 |
| S6 | TI Asthma OR TI Asthmas OR TI Bronchial Asthma OR TI Asthma, Bronchial | 4,117 |
| S5 | (MH "Asthma") | 0 |
| S4 | S1 OR S2 OR S3 | 3,028 |
| S3 | AB Chronic Obstructive Lung Disease OR AB Chronic Obstructive Pulmonary Diseases OR AB COAD OR AB COPD OR AB Chronic Obstructive Airway Disease OR AB Chronic Obstructive Pulmonary Disease OR AB Airflow Obstruction, Chronic OR AB Airflow Obstructions, Chronic OR AB Chronic Airflow Obstructions OR AB Chronic Airflow Obstruction | 2,973 |
| S2 | TI Chronic Obstructive Lung Disease OR TI Chronic Obstructive Pulmonary Diseases OR TI COAD OR TI COPD OR TI Chronic Obstructive Airway Disease OR TI Chronic Obstructive Pulmonary Disease OR TI Airflow Obstruction, Chronic OR TI Airflow Obstructions, Chronic OR TI Chronic Airflow Obstructions OR TI Chronic Airflow Obstruction | 1,389 |
| S1 | (MH "Pulmonary Disease, Chronic Obstructive") | 0 |

**Supplementary file 1.6: The Cochrane Library database search strategy**

| ID | Search | Hits |
| --- | --- | --- |
| #1 | MeSH descriptor: [Nebulizers and Vaporizers] explode all trees | 2621 |
| #2 | (Vaporizer*):ti,ab,kw | 2352 |
| #3 | (Inhaler*):ti,ab,kw | 7239 |
| #4 | (Inhalator*):ti,ab,kw | 186 |
| #5 | (Nebulizer*):ti,ab,kw | 3667 |
| #6 | (Atomizer*):ti,ab,kw | 102 |
| #7 | (Inhalation Devices):ti,ab,kw | 3225 |
| #8 | (Device, Inhalation):ti,ab,kw | 3227 |
| #9 | (Devices, Inhalation):ti,ab,kw | 3225 |
| #10 | (Inhalation Device):ti,ab,kw | 3227 |
| #11 | #1 OR #2 OR #3 OR #4 OR #5 OR #6 OR #7 OR #8 OR #9 OR #10 | 11742 |
| #12 | MeSH descriptor: [Administration, Inhalation] explode all trees | 6111 |
| #13 | (Drug Administration, Respiratory):ti,ab,kw | 17154 |
| #14 | (Administration, Respiratory Drug):ti,ab,kw | 17154 |
| #15 | (Respiratory Drug Administration):ti,ab,kw | 17154 |
| #16 | (Inhalation Drug Administration):ti,ab,kw OR (Drug Administration, Inhalation):ti,ab,kw OR (Administration, Inhalation Drug):ti,ab,kw OR (Inhalation Administration):ti,ab,kw OR (Drug Aerosol Therapy):ti,ab,kw | 19809 |
| #17 | (Aerosol Therapy, Drug):ti,ab,kw OR (Therapy, Drug Aerosol):ti,ab,kw OR (Drug Therapy, Aerosol):ti,ab,kw OR (Aerosol Drug Therapy):ti,ab,kw OR (Therapy, Aerosol Drug):ti,ab,kw | 3346 |
| #18 | (Inhalation of Drugs):ti,ab,kw | 22299 |
| #19 | #12 OR #13 OR #14 OR #15 OR #16 OR #17 OR #18 | 40495 |
| #20 | #11 OR #19 | 44859 |
| #21 | (sensor*):ti,ab,kw | 30929 |
| #22 | (passive monitoring):ti,ab,kw OR (inhaler monitoring):ti,ab,kw OR (electronic medication monitor):ti,ab,kw OR (electronic medication):ti,ab,kw OR (monitoring):ti,ab,kw | 130152 |
| #23 | (monitoring sensors):ti,ab,kw OR (medication monitoring):ti,ab,kw | 30914 |
| #24 | #21 OR #22 OR #23 | 156705 |
| #25 | #20 AND #24 | 5778 |
| #26 | (Nebulizer Chronolog):ti,ab,kw OR (MDI Chronolog):ti,ab,kw OR (Aerosol Actuation Counter):ti,ab,kw OR (Turbuhaler Inhalation):ti,ab,kw OR (Electronic Diskhaler):ti,ab,kw | 817 |
| #27 | (SmartMist):ti,ab,kw OR (MDILog):ti,ab,kw OR (Smart Inhaler Tracker):ti,ab,kw OR (Diskus Adherence Logger):ti,ab,kw OR (SmartTrack):ti,ab,kw | 13 |
| #28 | (SmartDisk):ti,ab,kw OR (SmartTurbo):ti,ab,kw OR (SmartFlow):ti,ab,kw OR (SmartMat):ti,ab,kw OR (Inhaler Compliance Assessment Device):ti,ab,kw | 135 |
| #29 | (Asthmapolis):ti,ab,kw OR (Propeller Health):ti,ab,kw OR (Chameleon):ti,ab,kw OR (SmartTouch):ti,ab,kw OR (MDI Acoustic Actuation Detector):ti,ab,kw | 188 |
| #30 | #26 OR #27 OR #28 OR #29 | 1140 |
| #31 | #25 OR #30 | 6791 |
| #32 | (Research, Qualitative):ti,ab,kw OR (Qualitative Research):ti,ab,kw | 10786 |
| #33 | MeSH descriptor: [Qualitative Research] explode all trees | 2082 |
| #34 | (Focus Group):ti,ab,kw OR (Group, Focus):ti,ab,kw OR (Groups, Focus):ti,ab,kw | 37856 |
| #35 | MeSH descriptor: [Focus Groups] explode all trees | 917 |
| #36 | #33 OR #32 | 10787 |
| #37 | #35 OR #34 | 37856 |
| #38 | (Interviews, Telephone):ti,ab,kw OR (Interview, Telephone):ti,ab,kw OR (Telephone Interview):ti,ab,kw OR (Telephone Interviews):ti,ab,kw OR (Group Interviews):ti,ab,kw | 32142 |
| #39 | MeSH descriptor: [Interview] explode all trees | 822 |
| #40 | (Group Interview):ti,ab,kw OR (Interview, Group):ti,ab,kw OR (Interviews, Group):ti,ab,kw OR (Interviewers):ti,ab,kw OR (Interviewer):ti,ab,kw | 49147 |
| #41 | #39 OR #40 | 49147 |
| #42 | MeSH descriptor: [Narration] explode all trees | 282 |
| #43 | (Narrations):ti,ab,kw OR (Narrative Ethics):ti,ab,kw OR (Ethics, Narrative):ti,ab,kw OR (Narration):ti,ab,kw | 3456 |
| #44 | #42 OR #43 | 3456 |
| #45 | (Grounded Theory):ti,ab,kw OR (Theory, Grounded):ti,ab,kw | 846 |
| #46 | MeSH descriptor: [Grounded Theory] explode all trees | 41 |
| #47 | #46 OR #45 | 846 |
| #48 | (case study):ti,ab,kw OR (content analysis):ti,ab,kw OR (descriptive study):ti,ab,kw OR (phenomenology):ti,ab,kw OR (thematic analysis):ti,ab,kw | 190380 |
| #49 | (action research):ti,ab,kw | 8898 |
| #50 | #36 OR #37 OR #41 OR #44 OR #47 OR #48 OR #49 | 265864 |
| #51 | #31 AND #50 | 1158 |
| #52 | (Chronic Obstructive Lung Disease):ti,ab,kw OR (Chronic Obstructive Pulmonary Diseases):ti,ab,kw OR (COAD):ti,ab,kw OR (COPD):ti,ab,kw OR (Chronic Obstructive Airway Disease):ti,ab,kw (Word variations have been searched) | 24900 |
| #53 | MeSH descriptor: [Pulmonary Disease, Chronic Obstructive] explode all trees | 7317 |
| #54 | (Chronic Obstructive Pulmonary Disease):ti,ab,kw OR (Airflow Obstruction, Chronic):ti,ab,kw OR (Airflow Obstructions, Chronic):ti,ab,kw OR (Chronic Airflow Obstructions):ti,ab,kw OR (Chronic Airflow Obstruction):ti,ab,kw (Word variations have been searched) | 16934 |
| #55 | #53 OR #52 OR #54 | 25304 |
| #56 | (Asthma):ti,ab,kw OR (Asthmas):ti,ab,kw OR (Bronchial Asthma):ti,ab,kw OR (Asthma, Bronchial):ti,ab,kw AND (asthma):ti,ab,kw (Word variations have been searched) | 35249 |
| #57 | MeSH descriptor: [Asthma] explode all trees | 15057 |
| #58 | #57 OR #56 | 35257 |
| #59 | #55 OR #58 | 57995 |
| #60 | #51 AND #59 | 347 |
