# Supplementary material for: Experience of Using Electronic Inhaler Monitoring Devices for Patients With Chronic Obstructive Pulmonary Disease or Asthma: Systematic Review of Qualitative Studies
Source: JMIR Mhealth Uhealth. 2025 May 16;13:e57645. doi: 10.2196/57645 (PMC12101605; doi:10.2196/57645)
Supplement: Multimedia Appendix 2 [file mhealth-v13-e57645-s002.doc]

**Multimedia Appendix 2**

**Quality Assessment Form for Included Studies**

| **Studies** | **Items** | | | | | | | | | |
| --- | --- | --- | --- | --- | --- | --- | --- | --- | --- | --- |
|  | **①** | **②** | **③** | **④** | **⑤** | **⑥** | **⑦** | **⑧** | **⑨** | **⑩** |
| **Van^[20]^** | Yes | Yes | Yes | Yes | Yes | No | No | Yes | Yes | Yes |
| **Hesso^[26]^** | Yes | Yes | Yes | Yes | Yes | No | Yes | Yes | Yes | Yes |
| **Hui^[27]^** | Yes | Yes | Yes | Yes | Yes | No | Yes | Yes | Yes | Yes |
| **Adejumo^[21]^** | Yes | Yes | Yes | Yes | Yes | Yes | Yes | Yes | Yes | Yes |
| **Jácome^[28]^** | Yes | Yes | Yes | Yes | Unclear | No | Yes | Yes | Yes | Yes |
| **Foster^[29]^** | Yes | Yes | Yes | Yes | Yes | No | Yes | Yes | Yes | Yes |
| **Kayyali^[30]^** | Yes | Yes | Yes | Yes | Yes | No | No | Yes | Yes | Yes |

Note:①Was there a clear statement of the aims of the research? Is a qualitative methodology appropriate? ③Was the research design appropriate to address the aims of the research? ④Was the recruitment strategy appropriate to the aims of the research? ⑤Was the data collected in a way that addressed the research issue? ⑥Has the relationship between the researcher and participants been adequately considered? ⑦Have ethical issues been taken into consideration? ⑧Was the data analysis sufficiently rigorous? ⑨Is there a clear statement of findings? ⑩How valuable is the research?
